# N-Heterocyclic Carbene Silver Complex Modified Polyacrylonitrile Fiber/MIL-101(Cr) Composite as Efficient Chiral Catalyst for Three-Component Coupling Reaction

**DOI:** 10.3390/nano12234175

**Published:** 2022-11-24

**Authors:** Ningning Xin, Xuemin Jing, Cheng-Gen Zhang, Xiaoxia Peng, Jing Liu, Qixing Wang, Wei Wang, Jian Cao, Minli Tao

**Affiliations:** 1 School of Chemistry and Material Science, Langfang Normal University, Langfang 065000, China; 2 Department of Chemistry, School of Science, Tianjin University, Tianjin 300072, China; 3 Hebei Diyuan Pharmaceutical Technology Co., Ltd., Cangzhou 061007, China

**Keywords:** carbenes, coupling reaction, nanoparticles, polyacrylonitrile fiber, metal-organic framework

## Abstract

Complex asymmetric synthesis can be realized by the chiral induction of amino acids in nature. It is of great significance to design a new biomimetic catalytic system for asymmetric synthesis. In this context, we report the preparation and characterization of the composite of polyacrylonitrile fiber (PANF) and metal-organic framework to catalyze the chiral synthesis of propargylamines. A confined microenvironment is established with N-heterocyclic carbene (NHC) silver complex-supported PANF and D-proline-encapsulated MIL-101(Cr). This novel supported catalyst demonstrated high activity in addition to excellent stereoselectivity in the three-component reaction between alkynes, aldehydes, and amines (A3). The regeneration can be realized by adsorption of D-proline again when the stereoselectivity decreases after recycle uses. By regulating the confined microenvironment on the composite, the activity and selectivity of the catalytic system are improved with turnover numbers of up to 2800 and 98% ee. The biomimetic catalytic system to A3 coupling reaction is systematically studied, and the synergistic catalytic mechanism between NHC-Ag and D-proline in the confined microenvironment is revealed.

## 1. Introduction

Propargylamine is a chiral nitrogenous compound with important physiological activity, and its structural unit exists in many natural and synthetic drugs [1,2]. The three-component coupling reaction of aldehydes, amines, and alkynes (A3 reaction) is a fascinating green method for the preparation of propargyl amine [3]. The A3 reaction produces a chiral center. A lot of researches have been carried out on the enantioselective A3 reaction [4,5]. In 2006, Singh et al. [6] reported that CuPF_6_ as a metal catalyst can catalyze the A3 reaction asymmetrically at room temperature. The catalytic system is suitable for a variety of substrates, and the product ee value can reach 99%. In 2010, Nakamura et al. [7] used (CuOTf)_2_·toluene as a metal catalyst and Pybim-based chiral bisimidazoline as a ligand for asymmetric A3 reaction of aromatic primary amines. In 2014, the team improved the catalytic system by optimizing the Pybim ligand and replacing the benzoyl group with the tert-butyryl group. At the same time, the asymmetric A3 reaction of aromatic amines in aqueous phase was successfully realized by the addition of sodium surfactant sulfate [8]. In 2015, Zhao et al. [9] reported a novel ligand containing thiourea and trans-1,2-cyclohexanediamine structure to induce CuI to catalyze the A3 reaction asymmetrically.

The supported catalyst has the advantages of high catalytic activity, good stability, and good repeatability. As a result, reports of multiphase A3 reactions with metal catalysts immobilized on supports have continued to emerge [10,11,12]. In 2008, Wang et al. reported the use of silver azacarbene functionalized polystyrene to catalyze the A3 reaction, and the activity of the catalyst did not decrease after 12 cycles [10]. Corma et al. [13] immobilized Au(III) on CeO_2_ and ZrO_2_ nanoparticles to catalyze the A3 reaction. The TON catalyzed by AuCl_3_ before immobilization was 273, while the TON after immobilization reached 10,760, which was 50 times higher than before. In 2012, Cai et al. [14] immobilized azacarbene silver functional groups on polystyrene polymers to catalyze the A3 reaction by the Click reaction. Iglesias et al. [15] immobilized a series of azacarbene gold complexes on MCM-41 molecular sieve, and the A3 reaction was catalyzed efficiently with a maximum yield of 97%. 

As a kind of very effective ligand, nitrogen heterocyclic carbene (N-heterocyclic carbene, NHC) plays an important role in the field of organic chemistry [16,17,18]. In 1995, the Hemnann team first reported the application of nitrogen heterocyclic carbene transition metal compounds in catalytic reactions [19]. Since then, many highly active and selective carbene metal complex catalysts have been synthesized and widely used in organic reactions [20,21]. Based on the above advantages, the development of different types of supported N-heterocyclic carbene catalysts and their application in the field of catalysis has become one of the research hotspots in organic chemistry [22]. The performance of supported N-heterocyclic carbene catalysts is not only related to the supported functional groups, but also related to the supported materials [23,24,25]. Polyacrylonitrile fiber (PANF) has a large specific surface area, an easy recovery, good flexibility, and a low price. Furthermore, there are a lot of cyano groups on the surface of acrylic fiber which have chemical activity and can be modified easily [26,27]. Li et al. [28] immobilized the Schiff base copper complex on the acrylic fiber, and the reaction of 1,3-dipolar cycloaddition (CuAAC) and A3 showed good stability and recoverability (>10 times). Shi et al. [29] reported a novel fiber-supported N-heterocyclic carbene (NHC) precursor and copper(I) composite catalyst for cleaner carboxylation of terminal alkynes from CO_2_. Xu et al. [30] synthesized a recyclable iron-loaded aminated polyacrylonitrile fiber (PAN(A)F-Fe) through a facile chemical grafting reaction for phosphate removal from wastewater. Therefore, the study on the synthesis, performance, and catalytic activity of acrylic fiber supported catalysts has high academic value and broad application prospects.

MOF has an attractive prospect in many fields, such as drug delivery [31], adsorption/separation processes [32,33], sensor [34,35], and supported catalysis [36,37]. MOFs have shown great potential because they exhibit many desirable properties of heterogeneous catalysts, such as crystallinity, uniformity of active sites, high surface area, and permanent porosity [38]. They can act as artificial enzymes to catalyze enantioselective heterogeneous reaction [39,40,41]. Among them, the easily accessible amino acids, especially proline, are usually used in MOFs catalyzed chiral reaction [42,43,44]. Telfer et al. reported IRMOFs [45] and MUF-77 [46] with L-proline linkers showed different selectivity. Kutzscher et al. reported a Boc-protected proline group was functionalized on the ligand of DUT-32 [47] and UiO-67/68 [48]. By employment of a chiral proline group, Chen et al. synthesized a defect-engineered chiral metal–organic framework with hierarchical micro/mesoporous structure for asymmetric aldol reaction [49]. Proline-functionalized MOFs MIL-53(Al) and DUT-5(Al) were synthesized by Janiak et al. to obtain advantageous increase of ee value [42]. 

**Figure 1:**
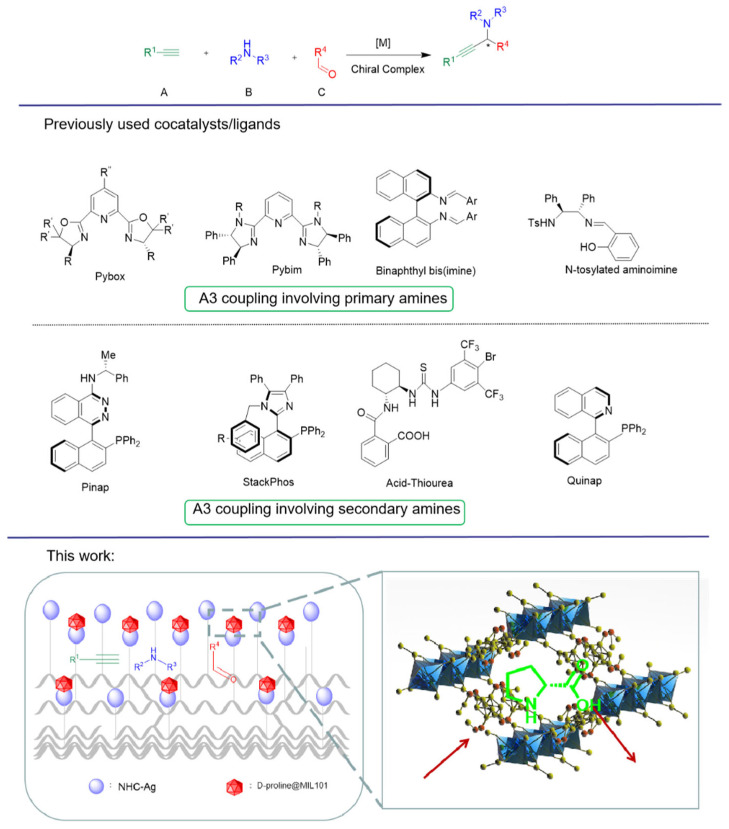
Stereoselective synthesis of propargylamines using A3 coupling reaction.

As mentioned above, there are asymmetric and immobilized catalysts for A3 reaction at present, but there are few reports on the immobilized chiral catalysts for A3 reaction. Due to the influence of intermolecular repulsion, different complicated chiral-induced ligands were used to bind well with the substrate, resulting in good stereoselectivity of A3 reaction (Figure 1) [5]. A good approach is to use naturally occurring chiral sources, such as chiral amino acid derivatives, to synthesize propargylamines. They are cheap and can be obtained in many forms. The ability of coupling metals to the amino acids with hydrogen bonds provides a simple way to induce enantioselectivity (via amino acids) and obtains the adjustability required for high selectivity (via the modification of metal catalysts). There are many metal-catalyzed reactions of substrates that can interact with amino acids. In this paper we construct a chiral-induced MOF-fiber composite to make molecules react in the microenvironment. Simple D-proline provides a way to introduce chirality into the MOF-fiber composite. A confined microenvironment is established with N-heterocyclic carbene (NHC) silver complex-supported PANF and D-proline-encapsulated MIL-101(Cr) (Figure 1). In addition, the modularization of the system allows it to be adjusted to obtain good enantioselectivity for A3 coupling reactions.

## 2. Materials and Methods

### 2.1. Materials

Commercially available chemical reactants were purchased from J&K Chemicals (Beijing, China), Bidepharm (Shanghai, China), and Beijing Ouhe (Beijing, China), and used without any further purification. All reagents were analytical grade. A commercially available PANF with a length of 10 cm and diameter of 30 ± 0.5 μm was purchased from the Fushun Petrochemical Corporation of China (Fushun, China). Column chromatography was performed over silica gel (200–300 mesh).

### 2.2. Preparation of the Fiber-MOFs Composite

The N-heterocyclic carbene silver complex modified polyacrylonitrile fiber (PANF_NHC_) was prepared as described in the Supplementary Materials (Scheme S1). PANF_NHC_ (1.0 g), chromium nitrate hexahydrate (2 mmol, 0.8 g), terephthalic acid (2 mmol, 0.32 g), and sodium hydroxide (5.0 mmol, 0.2 g) were charged to 50 mL of H_2_O and stirred for 30 min. All were sonicated and warmed in an autoclave at 393 K for 10 h. Then, the mixture was separated with centrifuge (3500 rpm, 20 min). The fiber-MOFs composite was cleaned with MeOH (10 mL). Finally, the fiber-MOFs composite was dried at 373 K in vacuum (1.12 g).

### 2.3. Encapsulation of D-Proline with Fiber-MOFs Composite

Fiber-MOFs composite (0.5 g) were added to 60 mL of *D*-proline in EtOH solution (100 mg mL^−1^). The mixture was stirred at 298 K for 1 d. The suspension was centrifuged (4000 rpm, 25 min), and the composited was cleaned with EtOH (5 mL × 2). The solid was dried under vacuum to obtain *D*-proline@PANF_NHC_-MIL101.

### 2.4. The Enantioselective A3 Reaction Catalyzed by PANF-MOFs Composite

Under N_2_, the *D*-proline@PANF_NHC_-MIL101 catalyst (56 mg), alkyne **1** (1.0 mmol, 1.0 equiv), aldehyde **2** (1.2 mmol, 1.2 equiv), and amine **3** (1.2 mmol, 1.2 equiv) were added to CH_3_CN (10 mL). The suspension was stirred at 273 K for 24 h. The reaction was motioned by HPLC, then the suspension was filtered and washed with CH_3_CN. Then, obtained solution was concentrated and then purified by flash chromatography (petroleum ether/ethyl acetate = 5:1) to obtain the products.

## 3. Results and Discussion

### 3.1. Preparation and Characterization of the MOFs-Fiber Composite

**Figure 2:**
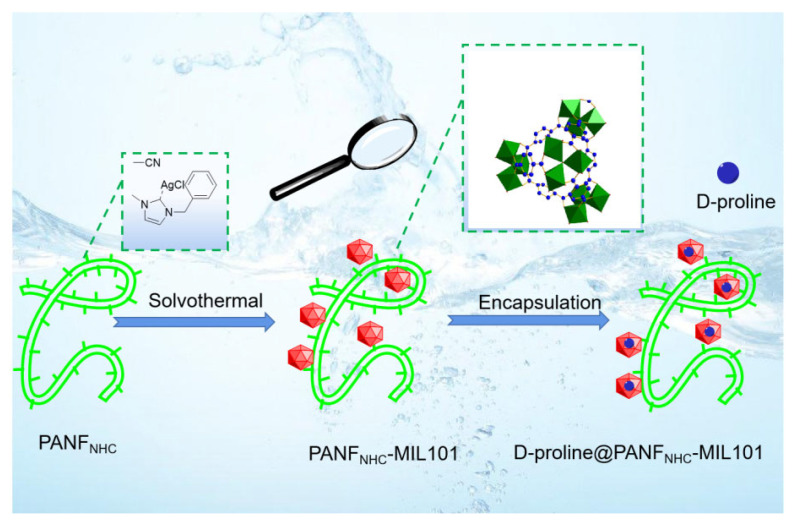
Schematic illustration of the preparation of D-proline@PANF_NHC_-MIL101.

**Figure 3:**
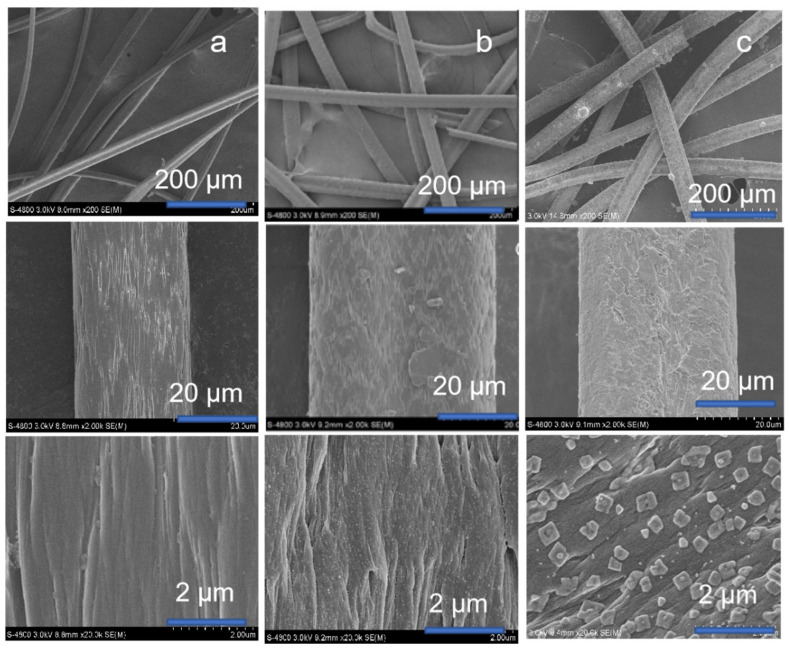
Scanning electron microscopy: (**a**) PANF, (**b**) PANF_NHC_, and (**c**) PANF_NHC_-MIL101.

As outlined in Figure 2, the preparation of *D*-proline@PANF_NHC_-MIL101 was based on the incorporation of D-proline into fiber-MOFs composite. In the first step, the PANF was modified to support Ag complexes [50]. Then, MIL101(Cr) was used to grow on fiber due to the framework’s good stability and uniformity of active sites for catalytic activity [51]. The MOFs-fiber composite is obtained by heating PANF and precursor of MOF. The nanoparticles of MOF coatings were observed on the surface of PANF_NHC_ by SEM imaging (Figure 3).

**Figure 4:**
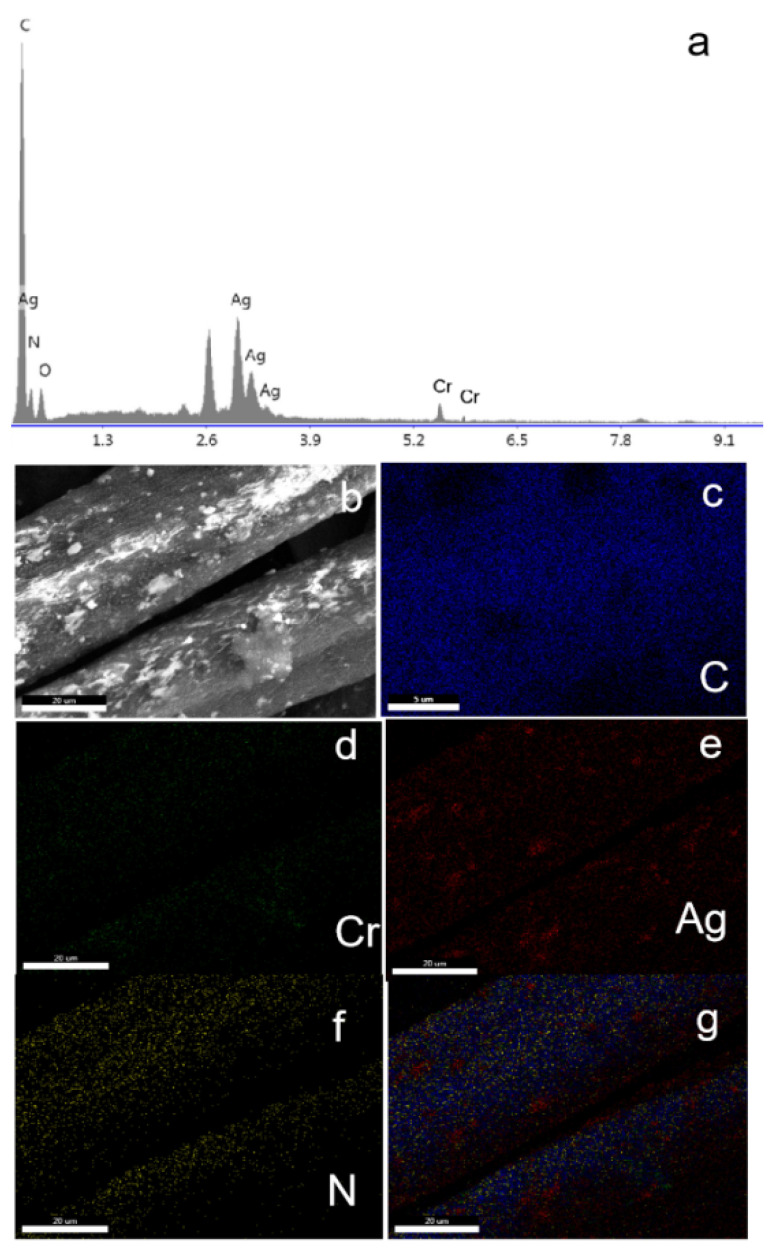
(**a**) EDS spectrum for PANF_NHC_-MIL101, (**b**) SEM image, and (**c**–**g**) energy-dispersive X-ray elemental mapping images for PANF_NHC_-MIL101.

The amount of its modification was determined by measuring the weight gain or the concentration of Ag by inductively coupled plasma optical emission spectroscopy (ICP-OES) (Table S1). The obtained PANF_NHC_ was then modified and coated with MIL101 by solvothermal method. As shown in Figure 3, the prepared PANF_NHC_ has a larger thickness and surface roughness than the PANF itself. In addition, following coating by MOFs, MIL-101(Cr) nanoparticles were found on the PANF_NHC_ surface. Subsequently, energy dispersive spectroscopy (EDS) was used to characterize the PANF_NHC_-MIL101. As shown in Figure 4a, peaks corresponding to C, N, Cr, and Ag were observed in EDS spectra to confirm that MIL-101(Cr) was anchored to the fiber surface. The prepared PANF_NHC_-MIL101 was examined by energy dispersive X-ray element mapping (Figure 4b–g). The highly dispersed MOFs in PANF matrix was the highlight. The element mapping also confirmed the dispersion of Cr, Ag, C, and O on the surface of the fiber.

**Figure 5:**
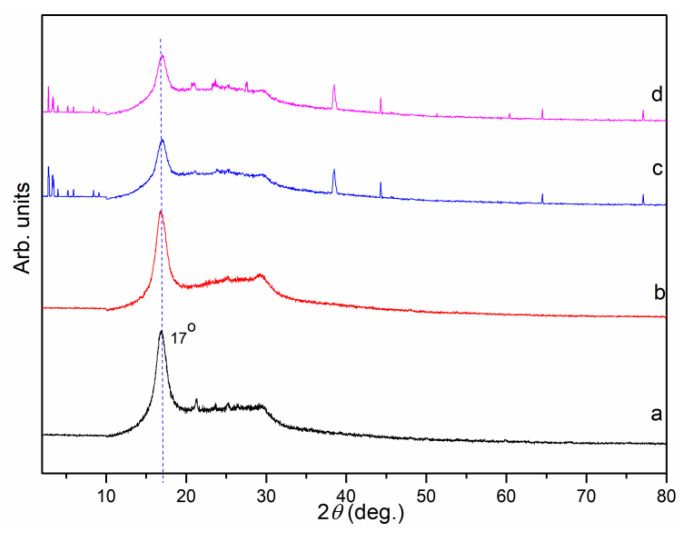
XRD patterns of the samples: (**a**) fiber, (**b**) fiber m with NHC Silver Complex, (**c**) PANF_NHC_-MIL101, and (**d**) *D*-proline@PANF_NHC_-MIL101.

The high quality MIL101(Cr) coatings were determined by PXRD. X-ray diffraction of the original PANF (Figure 5a) showed a strong reflection peak at 2*θ* = 17°, which was attributed to the (100) diffraction of the hexagonal lattice, which was constructed by the parallel close packing of the molecular rods [30]. For PANF_NHC_-MIL101 (Figure 5c), additional peaks at 2*θ* = 38.5, 44.3, 64.5, and 77.1° were ascribed to the (111), (200), (220), and (311) planes, respectively, which correspond to the structure of the MIL-101(Cr). Therefore, the results confirmed that Mil-101(Cr) were successfully coated on the surface of PANF [31].

N_2_ adsorption–desorption experiments for the composites proved that the coating of the highly porous MIL-101(Cr) (Table S2). Brunauer–Emmett–Teller (BET) surface area of PANF_NHC_-MIL101 increased from 55 to 182 m^2^/g when MIL-101 was coated on the surface of PANF_NHC_. By adjusting the solvothermal reaction conditions, the coating amount of the MIL-101(Cr) on PANF_NHC_ can be controlled. ICP-OES revealed that the coating amount ranged from 1.2 to 4.5% for Cr. The composite with 3% of Cr is selected because it has the largest specific surface area and is beneficial to catalysis. Moreover, the nanoparticles remained even when the composite was washed vigorously, due to strong intercrystalline interactions and strong bonds between the MOFs and the fibers [52]. 

### 3.2. Encapsulation of D-Proline with Fiber-MOFs Composite

Heterogeneous catalysis of porous materials is essential for the synthesis of many industrial chemicals [3]. The stereoselectivity of the amino-acid-catalyzed A3 reaction depends on the hydrogen-bonded cyclic transition state between the alkynes and enamine species [1]. MOFs tended to construct single-site solid catalysts with fascinating uniform catalytic sites and open channels for stereoselective reactions. Effective stereoselective catalysts based on MOFs must have large open channels to transport often very large organic substrates and products. This presents a major challenge, in part because MOFs constructed with slender bridging ligands tend to form interpenetrated structures that reduce or even eliminate the internal voids of the MOFs. In our study, MOF-fiber composites were prepared to avoid interpenetrating frames. Here, D-proline is encapsulated in fiber-MOFs composite to create a catalytically confined microenvironment [53]. With maximum optimization, the load of *D-*proline@PANF_NHC_-MIL101 was 4.7% *w*/*w*.

### 3.3. Chiral Catalytic Activity of the Fiber-MOFs in the A3 Coupling Reaction

Deprotonation of C_sp_–H to form C-C bonds is one of the most versatile and elegant operations in organic chemistry. The three component coupling reactions of aldehyde, amine, and alkyne (A3 coupling) for the synthesis of chiral propargylamine derivatives have attracted much attention due to its convenient approach, atom economy, and eco-friendly reaction condition [54,55]. However, there is still a need to develop a new highly stereoselective catalyst system for A3 coupling reactions [4,5].

Therefore, we first studied the catalytic performance of *D-*proline@PANF_NHC_-MIL101 in A3 coupling reaction, using benzaldehyde, 1-ethynyl-4-methylbenzene, and pyrrolidine as model reactants. The combination of PANF_NHC_ with *D*-proline promoted the preparation of propargylamine **3a** in 85% yield, although only 20% ee (Table S3, entry 1). Surprisingly, D-proline@PANFNHC-MIL101 significantly improved the enantioselectivity (Table S3, entry 2). Moreover, the highest ee was obtained in CH_3_CN, while other solvents, such as DMSO, THF, toluene, and DCM, afforded moderate-to-good stereoselectivity (Table S3, entries 5–9). Through the investigation of the reaction condition, 98% ee product **4a** was obtained in the reaction of 0 °C with 5 mol% of catalyst (entry 2). The feed ratio of terminal alkynes, aldehydes, and amines was optimized to give a ratio of 1.0:1.2:1.2.

The substrate range of the stereoselective A3 reaction was investigated under optimized conditions (Table 1). Aromatic aldehydes and terminal arylalkynes with different substituents were well tolerated. Whilst pyrrolidine performed as substrate, aromatic aldehydes with substituents in different ring positions all produced good results. More challenging secondary amines with aromatic ring were also good substrates. The lower selectivity was found by using aliphatic aldehydes. 

Then, the recoverability of *D-*proline@PANF_NHC_-MIL101 catalyst was studied. *D-*proline@PANF_NHC_-MIL101 was filtered out from the reaction mixture and reused in the subsequent entry to synthesize **4a**. After three cycles, the yield of **4a** was kept at 85%, but the ee decrease to 21%. Leaching of *D*-proline occurred after cycles, and it can be recovered by adsorption of *D*-proline again. The catalysis recoverability in the A3 reaction was investigated and good yield and high ee (97%) were obtained (Table S3 entry 11 and 12). When the catalyst loading was reduced to 0.01% and the reaction was carried out for 48 h, a 28% yield and an unprecedented 2800 cycles (TON) were obtained. 

### 3.4. Catalytic Reaction Pathway of the Supported Catalysts in the Three-Component Coupling Reaction

The oriented synthesis of chiral propargylamine has always been the focus and challenge in stereoselective reaction. In this project, functionalized ligands are immobilized to modify the fiber with high density and multi-layer. By coating with MOF, the confined microenvironment is bionically constructed on the fiber’s surface [37,44]. According to the selective NHC chelation of functionalized fibers, metal ions are immobilized on the fiber surface, and a bioinspired catalytic system with synergistic effect between metal-organic functionalized molecules and confined microenvironment is constructed [39]. By adjusting the structure of the ligand and appropriate metal, the chelation between ligand and metal and the activity of fiber-MOF composite are regulated correspondingly. By regulating the confined microenvironment [44], optimizing the reaction-transfer process, and utilizing the selective enrichment effect of fiber-MOF composite on substrates, the activity and selectivity of the catalytic system are improved [56]. 

**Figure 6:**
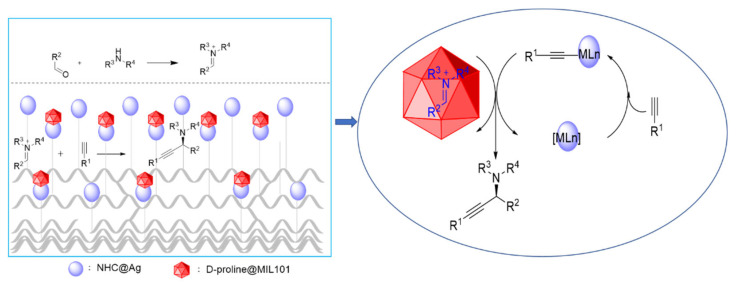
Proposed reaction pathway for the composite-catalyzed A3 reaction.

Figure 6 outlines the rational reaction pathway of A3 reaction catalyzed by *D*-proline@PANF_NHC_-MIL101 and suggests the possible approaches. The aldehyde interacts with the amine to produce an iminium ion intermediate, which reacts with the metal-alkynyl complex to form the desired product. The corresponding propargylamine can be synthesized from according imine and silver acetylide [36]. With a deep understanding of catalysis based on different MOFs, many mechanisms were proposed to explain the relationships between pore/channel and the catalysis ability [39]. Our result further supports that the decrease in catalytic activity during the additional runs might be due to pore blocking or blocking of the active site around the secondary building units (SBU) [39,40,44]. The bulkiness of the chiral molecule and its weak coordination to the metal ions afforded achiral structural isomers with discrete SBU. 

## 4. Conclusions

We herein reported the preparation and characterization of N-heterocyclic carbene silver complex modified polyacrylonitrile fiber/MIL-101(Cr) composite and the subsequent application for the efficient synthesis of chiral propargylamines. Stereoselective product **4a** (98% ee) was obtained, moreover the yield of **4a** was kept at 85% after three cycles. The stereoselectivity of the catalyst will decrease after recycle uses, and it can be regenerated by adsorption of D-proline again. By regulating the confined microenvironment, optimizing the reaction-transfer process, and utilizing the selective enrichment effect of fiber-MOF composite on substrates, the activity and selectivity of the catalytic system are improved. The catalytic A3 reaction of the biomimetic catalytic systems can systematically be studied, and the synergistic catalytic mechanism between chiral amino acid and the specific confined microenvironment of the composite is revealed. Importantly, these catalysts are easily accessible, and their modularity can be used to create a large number of different catalysts by using different members of the available pools of amino acids and N-heterocyclic carbene metal complex.

## Figures and Tables

**Table 1:** Stereoselective A3 reactions catalyzed by *D*-proline@PANF_NHC_-MIL101 ^a^.

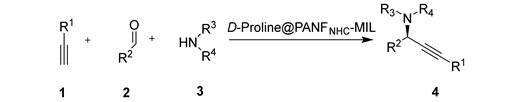
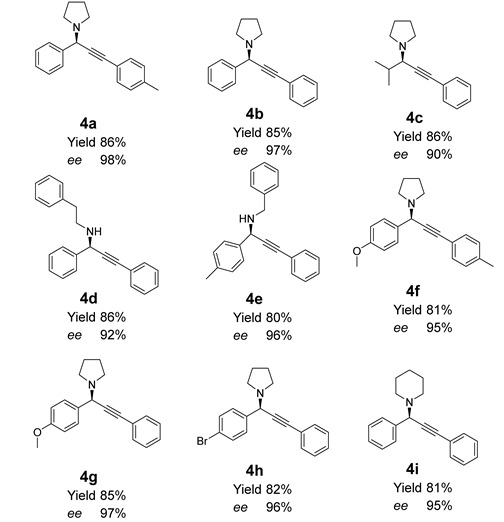

^a^ reaction condition: aldehydes (1.2 mmol), amines (1.2 mmol), alkynes (1.0 mmol), *D*-proline@PANF_NHC_-MIL101 (5 mol%, calculated based on Ag), 0 °C.

## Data Availability

The data presented in this study are available in this article.

## Supplementary Materials

The following supporting information can be downloaded at: https://www.mdpi.com/article/10.3390/nano12234175/s1, Scheme S1: Preparation of PANFNHC; Table S1: Different functionalities of PANFNHC measured by weight gains and metal contains; Table S2: The surface area and porosity of PANF and modified PANF; Table S3: Optimization studies for A3 coupling reaction; Characterization of the products; Figure S1: 1H NMR (400 MHz, CDCl3) spectrum of [N-benzyl-N’-(ethoxycarbonylmethyl)imidazolin-2-ylidene] silver chloride; Figure S2: 13C NMR (100 MHz, CDCl3) spectrum of [N-benzyl-N’-(ethoxycarbonylmethyl)imidazolin-2-ylidene]silver chloride; Figure S3: 1H NMR (400 MHz, CDCl3) spectrum of 1-(1-phenyl-3-(4-methylphenyl)-2-propynyl)pyrrolidine (4a); Figure S4: 13C NMR (100 MHz, CDCl3) spectrum of 1-(1-phenyl-3-(4-methylphenyl)-2-propynyl)pyrrolidine (4a); Figure S5: 1H NMR (400 MHz, CDCl3) spectrum of 1-(1,3-diphenyl-2-propynyl)pyrrolidine (4b); Figure S6: 13C NMR (100 MHz, CDCl3) spectrum of 1-(1,3-diphenyl-2-propynyl)pyrrolidine (4b); Figure S7: 1H NMR (400 MHz, CDCl3) spectrum of 1-(1-isopropyl-3-phenyl-2-propynyl)pyrrolidine (4c); Figure S8: 13C NMR (101 MHz, CDCl3) spectrum of 1-(1-isopropyl-3-phenyl-2-propynyl)pyrrolidine (4c); Figure S9: 1H NMR (400 MHz, CDCl3) spectrum of N-phenethyl-1,3-diphenylprop-2-yn-1-amine (4d); Figure S10: 13C NMR (100 MHz, CDCl3) spectrum of N-phenethyl-1,3-diphenylprop-2-yn-1-amine (4d); Figure S11: 1H NMR (400 MHz, CDCl3) spectrum of N-benzyl-3-phenyl-1-(p-tolyl)prop-2-yn-1-amine (4e); Figure S12: 13C NMR (101 MHz, CDCl3) spectrum of N-benzyl-3-phenyl-1-(p-tolyl)prop-2-yn-1-amine (4e); Figure S13: 1H NMR (400 MHz, CDCl3) spectrum of 1-(1-(4-methoxyphenyl)-3-(4-methylphenyl)-2-propynyl)pyrrolidine (4f); Figure S14: 13C NMR (100 MHz, CDCl3) spectrum of 1-(1-(4-methoxyphenyl)-3-(4-methylphenyl)-2-propynyl)pyrrolidine (4f)); Figure S15: 1H NMR (400 MHz, CDCl3) spectrum of 1-(1-(4-methoxyphenyl)-3-phenyl-2-propynyl)pyrrolidine (4g); Figure S16: 13C NMR (100 MHz, CDCl3) spectrum of 1-(1-(4-methoxyphenyl)-3-phenyl-2-propynyl)pyrrolidine (4g); Figure S17: 1H NMR (400 MHz, CDCl3) spectrum of 1-(1-(4-bromophenyl)-3-phenyl-2-propynyl)pyrrolidine (4h); Figure S18: 13C NMR (100 MHz, CDCl3) spectrum of 1-(1-(4-bromophenyl)-3-phenyl-2-propynyl)pyrrolidine (4h); Figure S19: 1H NMR (400 MHz, CDCl3) spectrum of 1-(1,3-diphenyl-2-propynyl)pyrrolidine (4i); Figure S20: 13C NMR (101 MHz, CDCl3) spectrum of 1-(1,3-diphenyl-2-propynyl)pyrrolidine (4i); Figure S21: HPLC Profile of 4a; Figure S22: HPLC Profile of 4b; Figure S23: HPLC Profile of 4c; Figure S24: HPLC Profile of 4d; Figure S25: HPLC Profile of 4e; Figure S26: HPLC Profile of 4f; Figure S27: HPLC Profile of 4g; Figure S28: HPLC Profile of 4h and Figure S29: HPLC Profile of 4i.
