# Supplementary material for: N-Heterocyclic Carbene Silver Complex Modified Polyacrylonitrile Fiber/MIL-101(Cr) Composite as Efficient Chiral Catalyst for Three-Component Coupling Reaction
Source: Nanomaterials (Basel). 2022 Nov 24;12(23):4175. doi: 10.3390/nano12234175 (PMC9736975; doi:10.3390/nano12234175)
Supplement: Supplementary file 1 [file nanomaterials-12-04175-s001.zip › nanomaterials-2023073-supplementary.pdf]

nanomaterials

Supplementary Materials

N-Heterocyclic Carbene Silver Complex Modified Polyacrylonitrile Fiber/MIL-101(Cr) Composite as Efficient Chiral Catalyst for Three-Component Coupling Reaction

Ningning Xin [1], Xuemin Jing [1], Cheng-Gen Zhang [1], Xiaoxia Peng [1], Jing Liu [1], Qixing Wang [2], Wei Wang [3], Jian Cao [1,*] and Minli Tao [2,*]

[1] School of Chemistry and Material Science, Langfang Normal University,Langfang 065000, China
[2] Department of Chemistry, School of Science, Tianjin University, Tianjin 300072, China
[3] Hebei Diyuan Pharmaceutical Technology Co. Ltd., Cangzhou 061007, China
* Correspondence: caojian@lfnu.edu.cn (J.C.); mltao@tju.edu.cn (M.T.)

1. General Experimental Information

1.1. Materials

A commercially available PANF with a length of 10 cm and diameter of 30 ± 0.5 μm was purchased from the Fushun Petrochemical Corporation of China. All reagents were analytical grade. Column chromatography was performed over silica gel (200–300 mesh).

1.2. Characterization and Measurements

The shapes and surface morphologies of the samples were observed with a Hitachi-S-4800 scanning electron microscope. D/MAX-2500 X-ray diffract meter (Hitachi, Japan) was performed to determine the crystallinity of the original and modified samples. An Elementar Vario EL instrument (Elementar, Germany) was used to perform the elemental analyses of the original and modified PANFs. ^{1}H NMR and ^{13}C NMR spectra were recorded on an AVANCE III spectrometer (Bruker, USA 400 MHz). Mass spectra were recorded on a miorOTOF-QII supplied by Bruker Daltonics (Bruker, USA). The concentrations of metal were determined using an ICP-9000 (N+M) inductively coupled plasma atomic emission spectrometer (ICP-AES, Thermo Jarrell-Ash, USA).

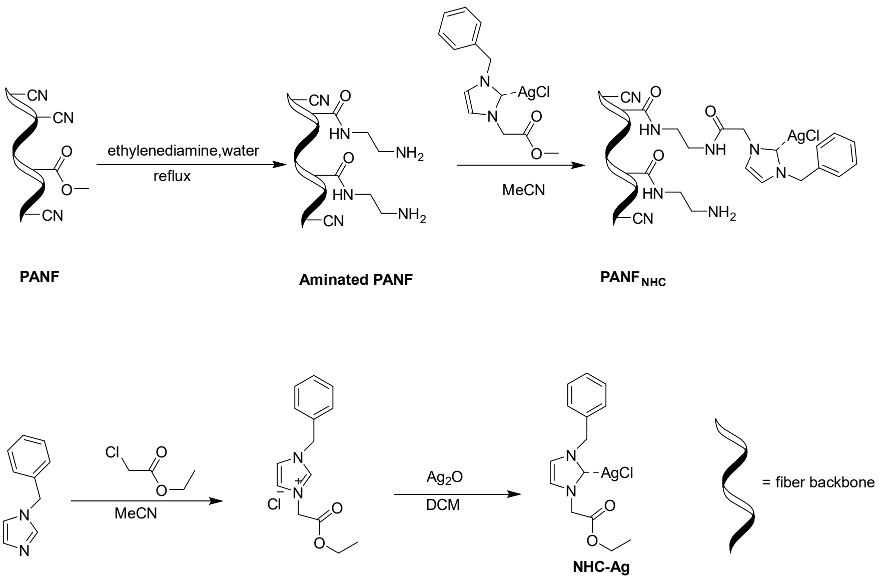

Scheme 1. Preparation of $PANF_{NHC}$.

1.3. Preparation of NHC-Ag

First 1-benzylimidazole (1.0 g, 6.3 mmol) and methyl chloroacetate (0.72 g, 6.6 mmol) were added to acetonitrile (20 mL) and refluxed for 12 h. The solvent was then evaporated to afford imidazolium salt as a yellow solid. Next, in an oven dried Schlenk flask, the imidazolium salt and silver(I) oxide (0.71 g, 3.2 mmol) were dissolved/suspended in dichloromethane (10 mL) and stirred at room temperature in the dark for 24 h. The resulting solution was filtered and concentrated, and the silver complex was obtained by recrystallization with EtOH and CH_2Cl_2 to afford 1.6 g of [*N*-benzyl-*N*′-(methoxycarbonyl methyl)imidazolin-2-ylidene]silver chloride (NHC-Ag).

1.4. Preparation of $PANF_{NHC}$

Dried PANF (5.0 g), deionized water (50 mL), and ethylenediamine (100 mL) was introduced into a 250-mL three-neck flask. The suspension was heated to reflux for 2 h. Next, the modified PANF was filtered out and washed with deionized water at 70–80 °C until the pH of the washed water was 7. Then the modified fiber was dried overnight under vacuum at 60 °C to give the aminated PANF. The weight gain of aminated PANF was 16.2% (functionality: 2.1 mmol/g).

NHC-Ag complex ([N-benzyl-N′-(methoxycarbonyl methyl) imidazolin-2-ylidene]silver chloride) was prepared as described in the supporting information. To a solution of NHC-Ag complex (3.2 g, 9.5 mmol) in CH_3CN (75 mL), ethylenediamine-aminated PANF (2.0 g, functionality: 2.1 mmol/g) was added, and the suspension was stirred at reflux for 4 h. The modified fiber was then filtered and washed with CH_3CN in a Soxhlet extractor for 24 h. Then the modified fiber was dried under vacuum to give $PANF_{NHC}$ (2.7 g, weight gain: 35%, Ag carbene functionality: 0.75 mmol/g).

1.5. Modification Extent

The extent of modification was determined by calculating the weight gain using the following equation: weight gain = [(W2 − W1)/W1] × 100%, where W1 and W2 are the weights of PANF and the modified fibers, respectively. The extent of modification was also measured by determining the concentration of metal ions by inductively coupled plasma optical emission spectroscopy (ICP-OES).

1.6. Encapsulation of D-Proline by $PANF_{NHC}$-MIL101

Fiber-MOFs composite (0.5 g) were stirred with 60 mL of *D*-proline (100 mg/mL) in EtOH under inert gas. The suspension was mixed at 25 °C for 24 h. Then, the mixture was centrifuged (3000 rpm, 15 min), and the obtained solid was washed and dried. After the desired absorption time, the residual concentrations (C_e) of *D*-proline were determined by high-performance liquid chromatography. The loading capacity (q_e, %) was calculated as follows,

$$q_e = \frac{(C_0 - C_e) \times V}{W} \quad (1)$$

where C_0 (mg/mL) is the initial *D*-proline concentration, C_e (mg/mL) is the equilibrium *D*-proline concentration, *V* is the solution volume (mL), and *W* is the composite weight (mg).

2. Supplementary Tables and Figures

Table S1. Different functionalities of $PANF_{NHC}$ measured by weight gains and metal contains.

Batch	1	2	3	4
Weight gain method (mmol/g)	0.21	0.43	0.75	1.1
Metal content method (mmol/g)	0.18	0.39	0.72	1.0

Feed ratio (NHC-Ag: Aminated PANF)	1:2	1:2	1.6:1	1.6:1
Reaction time	4 h	12 h	4 h	12 h

Table S2. The surface area and porosity of PANF and modified PANF.

	S_{BET}(m² g⁻¹)	V_{Tot}(cm³ g⁻¹)	D_{av}(nm)
PANF	41.1	0.034	3.27
Aminated PANF	33.1	0.143	3.18
$PANF_{NHC}$	55.1	0.228	3.41
$PANF_{NHC}$-MIL	182.3	1.66	3.21

Table S3. Optimization studies for A3 coupling reaction[a].

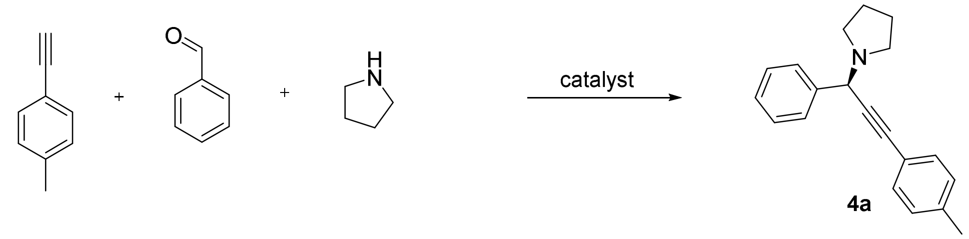

Entry	Catalyst	Solvent	Yield(%)	*ee* (%)	Temperature (°C)
1	$PANF_{NHC}$+*D*-proline[b]	CH_3CN	80	20	0
2	*D*-proline@$PANF_{NHC}$-MIL	CH_3CN	86	98	0
3	*D*-proline@$PANF_{NHC}$-MIL	CH_3CN	89	77	25
4	*D*-proline@$PANF_{NHC}$-MIL	CH_3CN	89	70	50
5	*D*-proline@$PANF_{NHC}$-MIL	toluene	80	82	0
6	*D*-proline@$PANF_{NHC}$-MIL	DMSO	78	85	0
7	*D*-proline@$PANF_{NHC}$-MIL	THF	72	78	0
8	*D*-proline@$PANF_{NHC}$-MIL	CH_2Cl_2	76	85	0
9	*D*-proline@$PANF_{NHC}$-MIL	EtOH	59	30	0
10	*D*-proline@$PANF_{NHC}$-MIL	neat	80	76	0
11	*D*-proline@$PANF_{NHC}$-MIL[c]	CH_3CN	84	21	0
12	*D*-proline@$PANF_{NHC}$-MIL[d]	CH_3CN	83	97	0

[a] reaction condition: benzaldehyde (1.2 mmol), pyrrolidine (1.2 mmol), 1-ethynyl-4-methylbenzene (1.0 mmol), *D*-proline@$PANF_{NHC}$-MIL (5 mol%, calculated based on Ag) and solvent (5 mL). [b] D-proline (5 mol%) $PANF_{NHC}$ (5 mol%, calculated based on Ag) and solvent (5 mL). [c] After 3 cycles. [d] After 3 cycles, the catalyst was recovered by adsorption of D-proline again.

3. Characterization of the Products

3.1. [. N-benzyl-N′-(ethoxycarbonylmethyl)imidazolin-2-ylidene]silver Chloride

White solid; yield: 75%; ^{1}H NMR (400 MHz, $CDCl_3$) δ = 7.37 – 7.27 (m, 3H), 7.25 – 7.23 (m, 2H), 7.09 (d, *J* = 3.8 Hz, 1H), 6.97 (d, *J* = 3.8 Hz, 1H), 5.30 (s, 2H), 4.93 (s, 2H), 4.27 (q, *J* = 7.8 Hz, 2H), 1.33 (t, *J* = 7.8 Hz, 3H); ^{13}C NMR (101 MHz, $CDCl_3$): δ = 167.30, 135.03, 129.23, 128.83, 127.82, 122.76, 121.51, 62.50, 55.93, 52.70,14.10; ESI-MS: *m*/*z* =246 (M-Ag-Cl+H)$^+$.

3.2. 1-(1-phenyl-3-(4-methylphenyl)-2-propynyl)pyrrolidine (4a)[1]

^{1}H NMR (400 MHz, $CDCl_3$) δ = 7.62 (t, J = 10.7 Hz, 2H), 7.45 – 7.26 (m, 5H), 7.14 (t, *J* = 8.9 Hz, 2H), 4.90 (s, 1H), 2.72 (d, *J* = 6.4 Hz, 4H), 2.37 (d, *J* = 5.7 Hz, 3H), 1.91 – 1.69 (m, 4H). ^{13}CNMR (100 MHz, $CDCl_3$): δ = 139.63, 138.14, 131.68, 129.01, 128.41, 128.31, 128.23, 127.52, 127.21, 120.19, 87.01, 85.91, 59.15, 50.25, 23.51, 21.45; ESI-MS: *m*/*z* = 276 (M+H)$^+$. Optical

rotation: $[\alpha]^{20}_{D}$ +16.5 (c 0.5, $CHCl_3$). HPLC (Chiral Pak OJ-H hexane/IPA= 95:5, flow rate=1 mL/min). The absolute configuration was assigned by analogy.

3.3. 1-(1,3-diphenyl-2-propynyl)pyrrolidine (4b)[2]

^{1}H NMR (400 MHz, $CDCl_3$) δ = 7.69-7.67 (m, 2H), 7.57-7.55 (m, 2H), 7.42-7.35 (m, 6H), 5.20 (s, 1H), 2.73-2.81 (m, 4H),1.87 (t, 4H, J=8.0 Hz); ^{13}C NMR (100 MHz, $CDCl_3$): δ = 140.01, 132.07, 128.90, 128.23, 127.52, 123.15, 86.62, 81.14, 54.73, 50.86, 26.35; ESI-MS: *m/z* = 284 $(M+Na)^+$.Optical rotation: $[\alpha]^{20}_{D}$ +8.3 (c 0.5, $CHCl_3$). HPLC (Chiral Pak OJ-H hexane/IPA= 95:5, flow rate=1 mL/min) The absolute configuration was assigned by analogy.

3.4. 1-(1-isopropyl-3-phenyl-2-propynyl)pyrrolidine (4c)

^{1}H NMR (400 MHz, $CDCl_3$): δ = 7.47-7.45 (m, 2H), 7.33-7.30 (m, 3H), 3.3-3.08 (m, 1H), 2.76-2.68 (m, 4H), 1.95-1.93 (m, 1H), 1.84-1.80 (m, 4H), 1.14 (d, *J* = 6.0 Hz, 3H), 1.08 (d, *J* = 6.0 Hz, 3H); ^{13}CNMR (100 MHz, $CDCl_3$): δ = 131.71, 128.19, 127.71, 123.68, 87.82, 85.59, 62.55, 50.35, 31.93, 23.55, 20.22, 19.46; ESI-MS: *m/z* = 228 $(M+H)^+$.Optical rotation: $[\alpha]^{20}_{D}$ +9.3 (c 0.5, $CHCl_3$). HPLC (Chiral Pak OJ-H hexane/IPA= 95:5, flow rate=1 mL/min) The absolute configuration was assigned by analogy.

3.5. N-phenethyl-1,3-diphenylprop-2-yn-1-amine (4d)

^{1}H NMR (400 MHz, $CDCl_3$) δ = 7.55 (d, J = 4.7 Hz, 2H), 7.44 (s, 2H), 7.35 – 7.19 (m, 11H), 4.83 (s, 1H), 3.14 – 3.11 (m, 1H), 3.04 – 3.01 (m, 1H), 2.88 – 2.83 (m, 2H), 1.63 (s, 1H). ^{13}CNMR (100 MHz, $CDCl_3$): 140.40, 139.95, 131.78, 128.81, 128.58, 128.52, 128.32, 128.21, 127.80, 127.64, 126.24, 123.18, 89.35, 85.49, 54.66, 48.43, 36.32; ESI-MS: *m/z* = 312 $(M+H)^+$.$[\alpha]^{20}_{D}$ +29.2 (c 0.5, $CHCl_3$). HPLC (Chiral Pak AD-H hexane/IPA= 95:5, flow rate=1 mL/min) The absolute configuration was assigned by analogy.

3.6. N-benzyl-3-phenyl-1-(p-tolyl)prop-2-yn-1-amine (4e)[3]

^{1}H NMR (400 MHz, $CDCl_3$) δ = 7.48 (m, 4H), 7.40 (m, 2H), 7.32 (m, 7H), 7.16 (m, 2H), 4.77 (s, 1H), 3.98 (s, 2H), 2.34 (s, 3H); ^{13}C NMR (100 MHz, $CDCl_3$): δ = 140.0, 137.5, 137.4 131.8, 129.2, 128.5, 128.4, 128.3, 128.2, 127.7, 127.1, 123.2, 89.5, 85.5, 53.5, 51.2, 21.1; ESI-MS: *m/z* = 312 $(M+H)^+$. Optical rotation: $[\alpha]^{25}_{D}$ +47.7 (c 0.5, $CHCl_3$). HPLC (Chiral Pak AD-H hexane/IPA= 95:5, flow rate=1 mL/min), The absolute configuration was assigned by analogy.

3.7. 1-(1-(4-methoxyphenyl)-3-(4-methylphenyl)-2-propynyl)pyrrolidine (4f)

^{1}H NMR (400 MHz, $CDCl_3$) δ = 7.55 (d, *J* = 8.6 Hz, 2H), 7.40 (d, *J* = 8.0 Hz, 2H), 7.14 (d, J = 7.9 Hz, 2H), 6.91 (d, *J* = 8.7 Hz, 2H), 4.89 (s, 1H), 3.83 (s, 3H), 2.74 (m, 4H), 2.37 (s, 3H), 1.84 (d, *J* = 6.1 Hz, 4H). ^{13}C NMR (100 MHz, $CDCl_3$) δ = 159.12, 138.19, 131.66, 129.49, 129.00, 120.10, 113.61, 86.99, 86.34, 58.51, 55.29, 50.20, 23.50, 21.44; ESI-MS: *m/z* = 306 $(M+H)^+$; HRMS (ESI): *m/z* $[M+H]^+$ calcd for $C_{21}H_{24}NO$: 306.1858, found: 306.1868. Optical rotation: $[\alpha]^{20}_{D}$ +12.3 (c 0.5, $CHCl_3$). HPLC (Chiral Pak OJ-H hexane/IPA= 95:5, flow rate=1 mL/min); The absolute configuration was assigned by analogy.

3.8. 1-(1-(4-methoxyphenyl)-3-phenyl-2-propynyl)pyrrolidine (4g)[1]

^{1}H NMR (400 MHz, $CDCl_3$) δ = 7.56 (d, *J* = 8.6 Hz, 2H), 7.51 (dd, *J* = 6.5, 2.9 Hz, 2H), 7.38 – 7.29 (m, 3H), 6.91 (d, *J* = 8.7 Hz, 2H), 4.96 (s, 1H), 3.83 (s, 3H), 2.78 (d, *J* = 5.2 Hz, 4H), 1.85 (s, 4H). ^{13}CNMR (100 MHz, $CDCl_3$): δ = 159.17, 131.79, 129.56, 128.28, 126.28, 123.17, 113.66, 87.00, 86.81, 58.42, 55.29, 50.16, 23.51; ESI-MS: *m/z* = 314 $(M+Na)^+$.Optical rotation: $[\alpha]^{20}_{D}$ +10.3 (c 0.5, $CHCl_3$). HPLC (Chiral Pak OJ-H hexane/IPA= 95:5, flow rate=1 mL/min); The absolute configuration was assigned by analogy.

3.9. 1-(1-(4-bromophenyl)-3-phenyl-2-propynyl)pyrrolidine (4h)[1]

^{1}H NMR (400 MHz, $CDCl_3$) δ = 7.53 – 7.49 (m, 6H), 7.47 – 7.29 (m, 3H), 4.91 (s, 1H), 2.71 (dd, J = 11.1, 6.2 Hz, 4H), 1.83 (t, *J* = 6.1 Hz, 4H). ^{13}C NMR (100 MHz, $CDCl_3$) δ = 138.51, 131.80, 131.36, 129.99, 128.29, 122.94, 121.52, 87.42, 85.81, 77.33, 77.02, 76.70, 58.39, 50.09, 23.53; ESI-MS: *m*/*z* = 340, 342 $(M+H)^+$.Optical rotation: $[\alpha]^{20}_{D}$ +13.5 (c 0.5, $CHCl_3$). HPLC (Chiral Pak OD-H hexane/IPA= 95:5, flow rate=1 mL/min); The absolute configuration was assigned by analogy.

3.10. 1-(1,3-diphenylprop-2-yn-1-yl)piperidine (4i)[2]

^{1}H NMR (400 MHz, $CDCl_3$) δ = 7.71 – 7.69 (m, 2H), 7.59 – 7.57 (m, 2H), 7.44 – 7.34 (m, 6H), 4.87 (s, 2H), 2.64 (bs, 4H), 1.68 – 1.64 (m, 4H), 1.52 – 1.51 (m, 2H). ^{13}C NMR (100 MHz, $CDCl_3$): δ = 138.63, 131.88, 128.61, 128.34, 128.12, 127.54, 123.43, 87.98, 86.13, 62.45, 50.76, 26.24, 24.52; ESI-MS: *m*/*z* = 298 $(M+Na)^+$. Optical rotation: $[\alpha]^{20}_{D}$ +5.6 (c 0.5, $CHCl_3$). HPLC (Chiral Pak AD-H hexane/IPA= 95:5, flow rate=1 mL/min); The absolute configuration was assigned by analogy.

4. NMR and HPLC Spectra for Selected Compounds

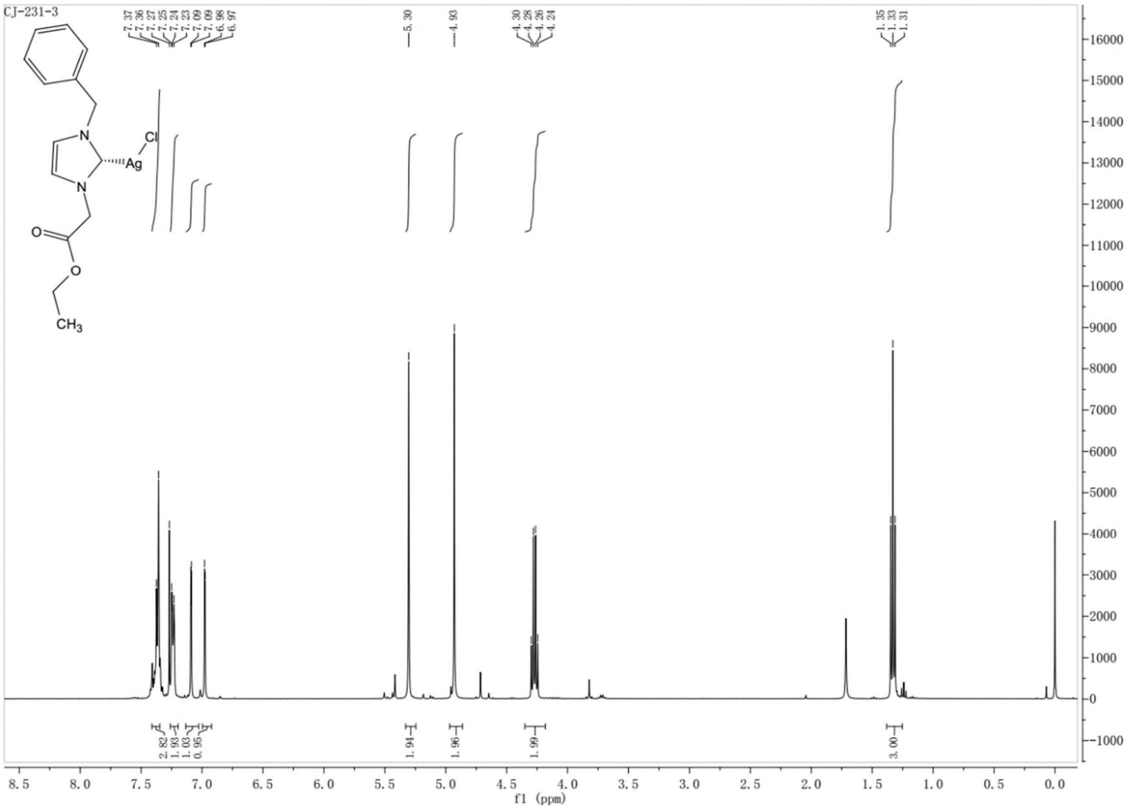

Figure S1. ^{1}H NMR (400 MHz, $CDCl_3$) spectrum of [*N*-benzyl-*N*'-(ethoxycarbonylmethyl)imidazolin-2-ylidene] silver chloride.

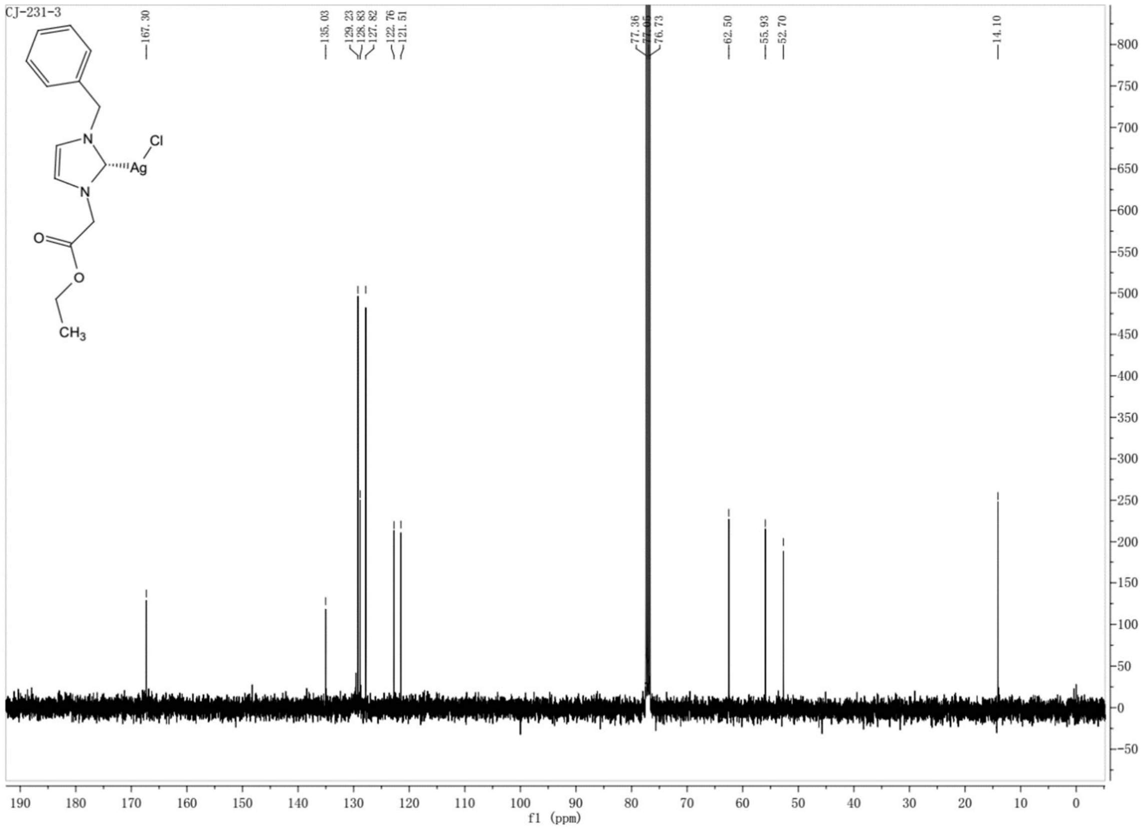

Figure S2. ^{13}C NMR (100 MHz, $CDCl_3$) spectrum of [*N*-benzyl-*N*′-(ethoxycarbonylmethyl)imidazolin-2-ylidene]silver chloride.

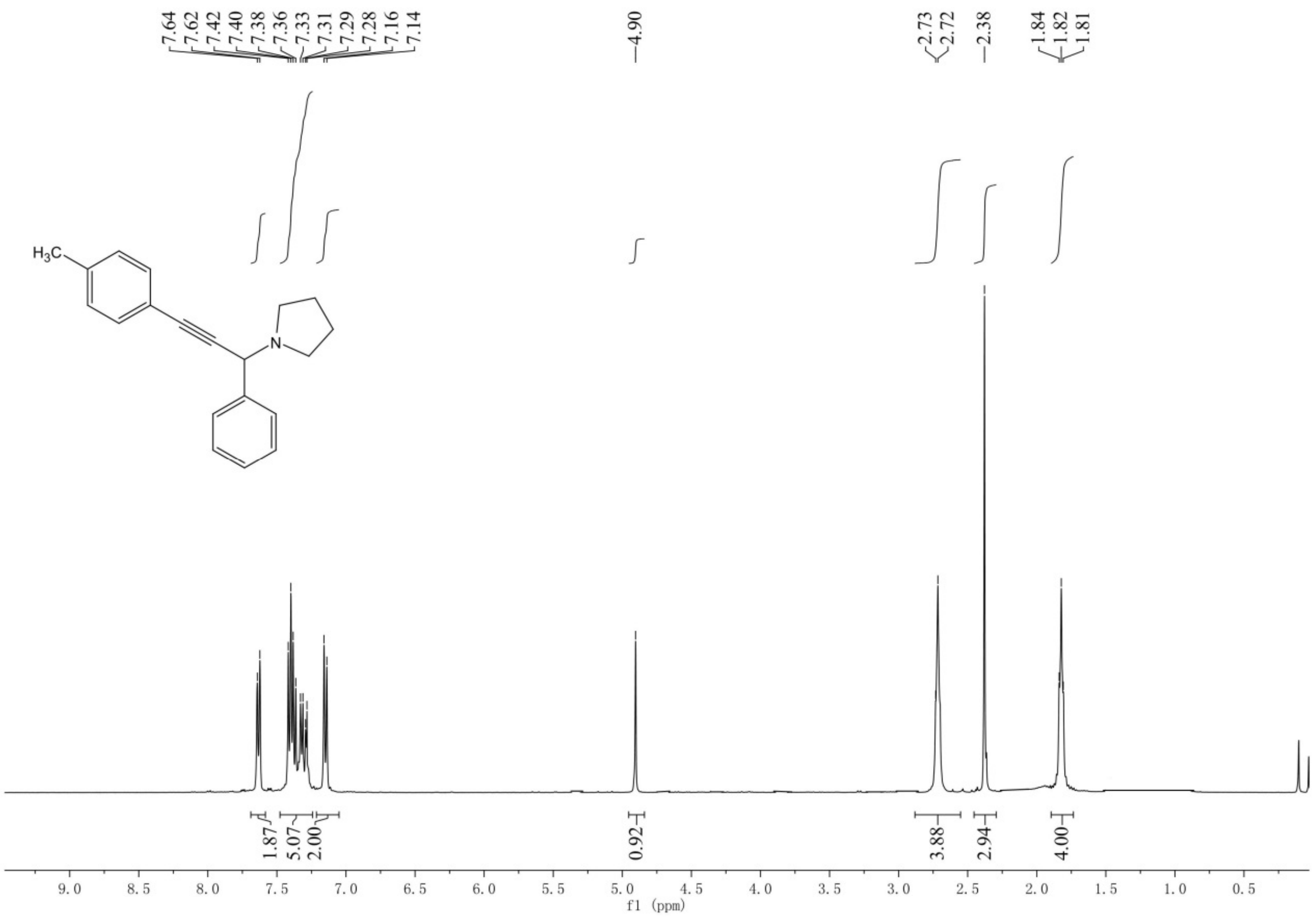

Figure S3. ^{1}H NMR (400 MHz, $CDCl_3$) spectrum of 1-(1-phenyl-3-(4-methylphenyl)-2-propynyl)pyrrolidine (4a).

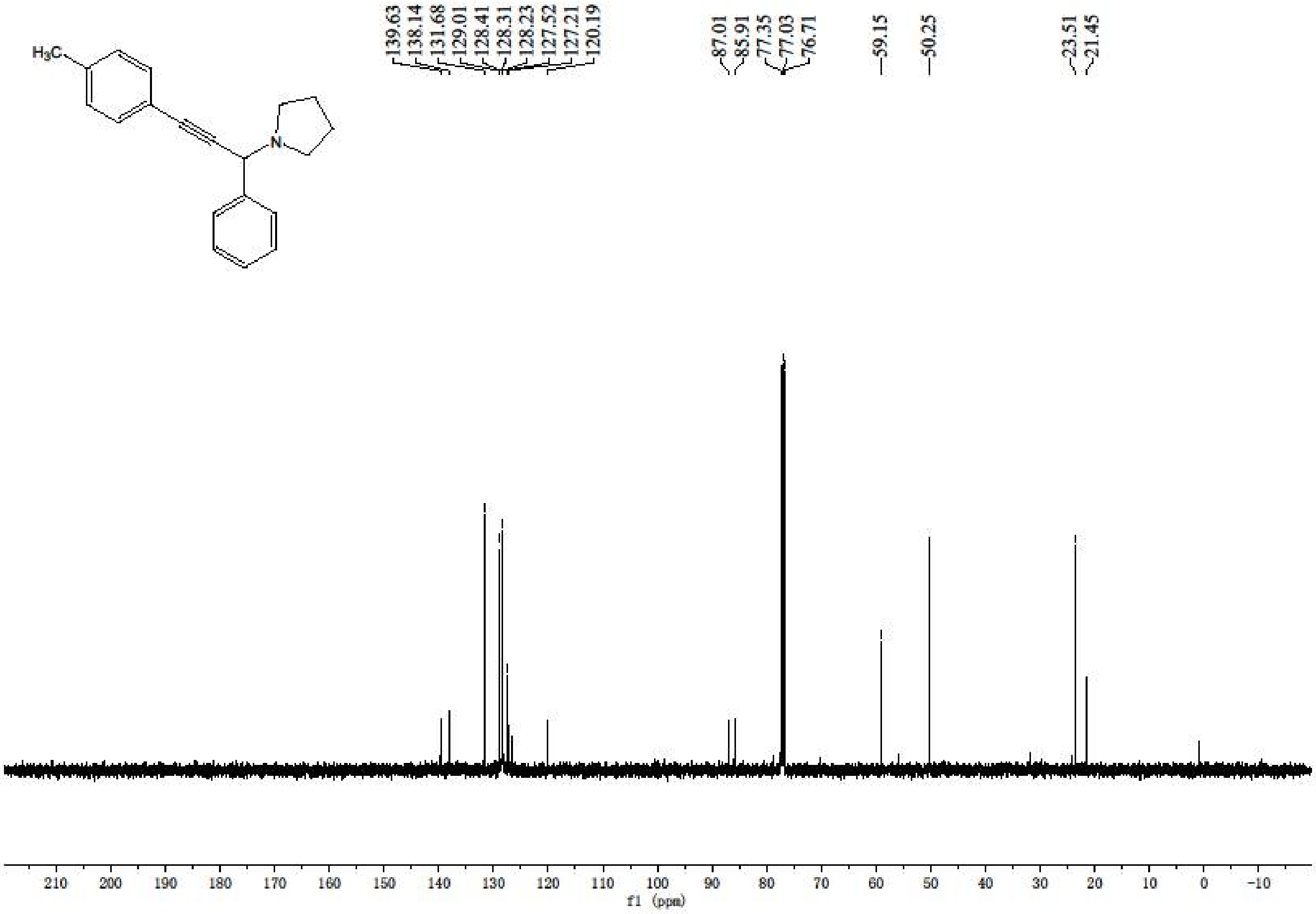

Figure S4. ^{13}C NMR (100 MHz, $CDCl_3$) spectrum of 1-(1-phenyl-3-(4-methylphenyl)-2-propynyl)pyrrolidine (4a).

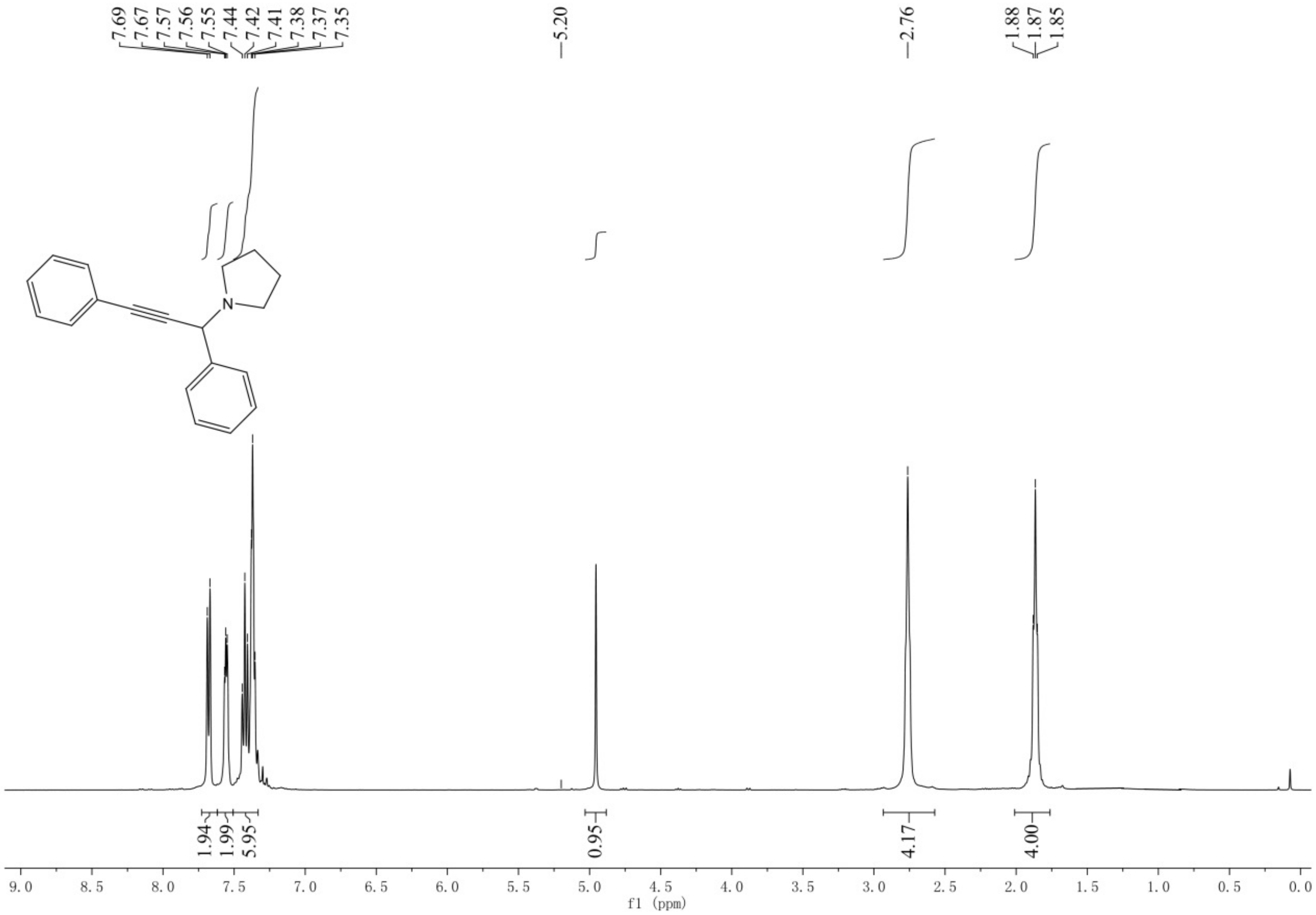

Figure S5. ^{1}H NMR (400 MHz, $CDCl_3$) spectrum of 1-(1,3-diphenyl-2-propynyl)pyrrolidine (4b).

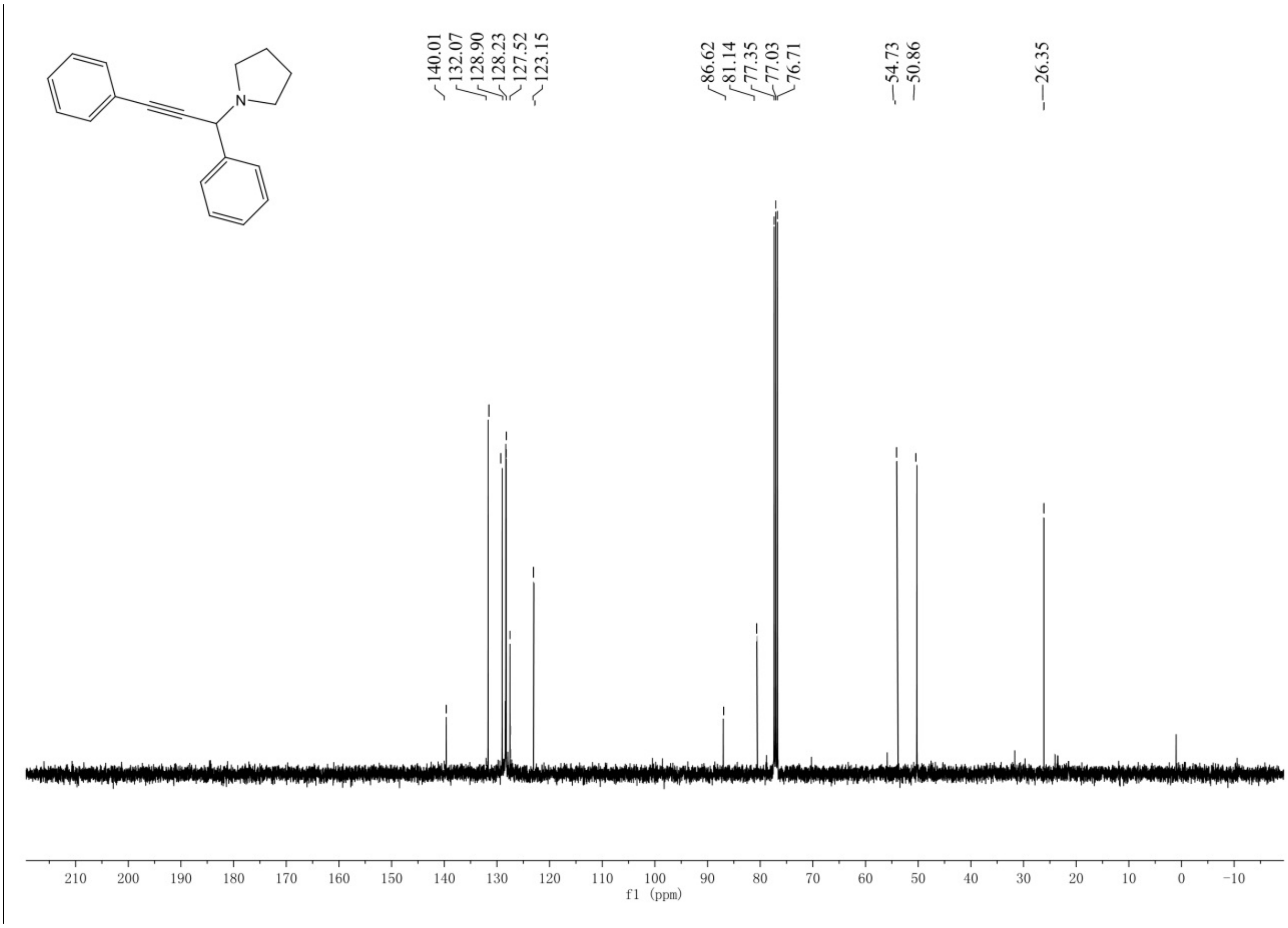

Figure S6. ^{13}C NMR (100 MHz, $CDCl_3$) spectrum of 1-(1,3-diphenyl-2-propynyl)pyrrolidine (4b).

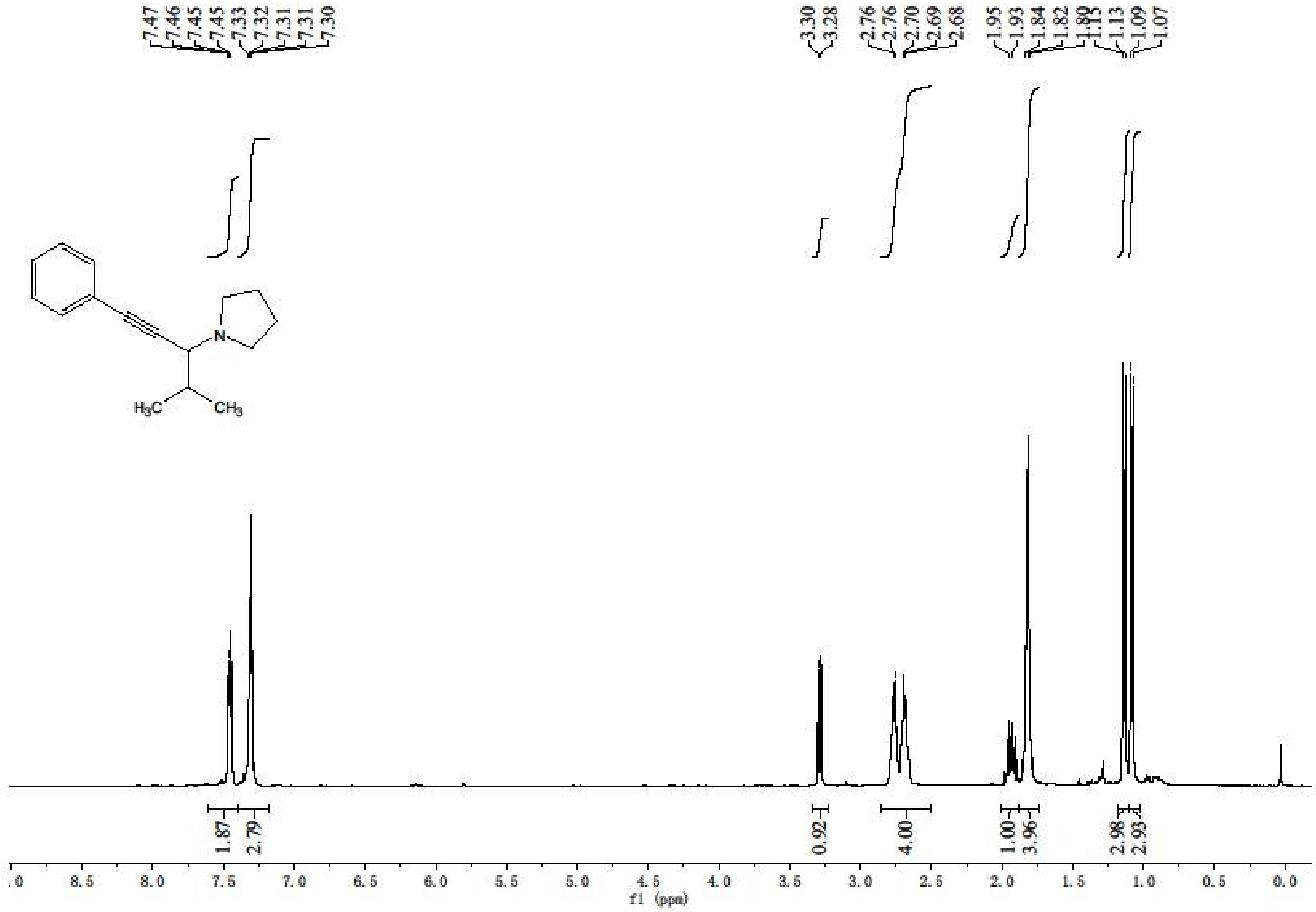

Figure S7. ^{1}H NMR (400 MHz, $CDCl_3$) spectrum of 1-(1-isopropyl-3-phenyl-2-propynyl)pyrrolidine (4c).

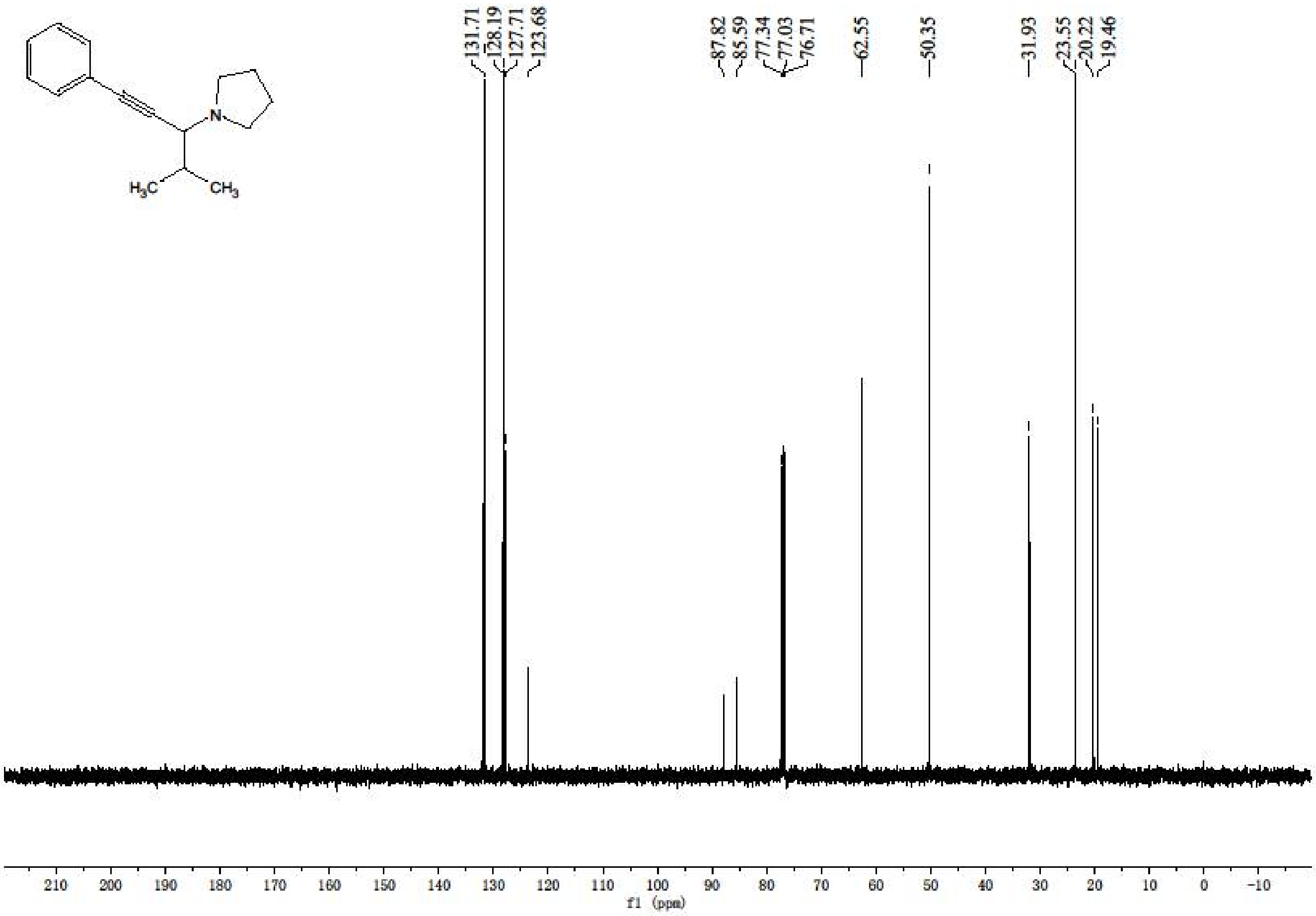

Figure S8. ^{13}C NMR (101 MHz, $CDCl_3$) spectrum of 1-(1-isopropyl-3-phenyl-2-propynyl)pyrrolidine (4c).

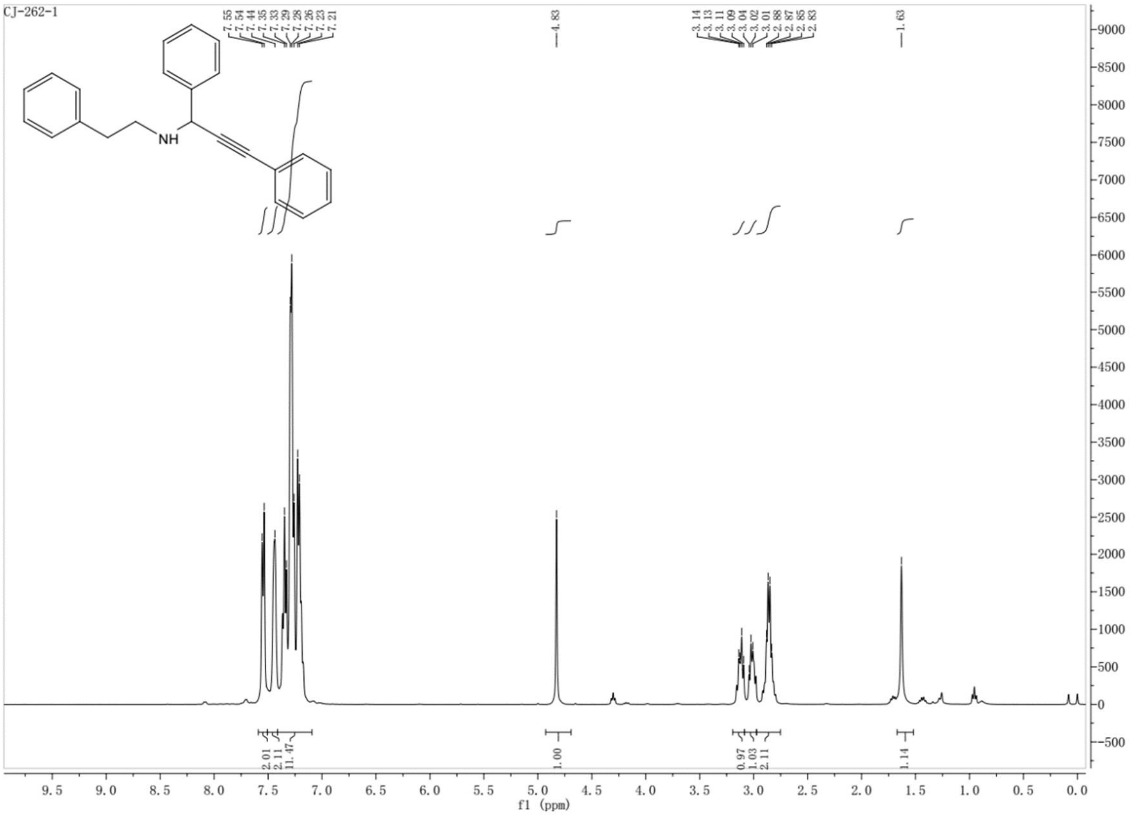

Figure S9. ^{1}H NMR (400 MHz, $CDCl_3$) spectrum of *N*-phenethyl-1,3-diphenylprop-2-yn-1-amine (4d).

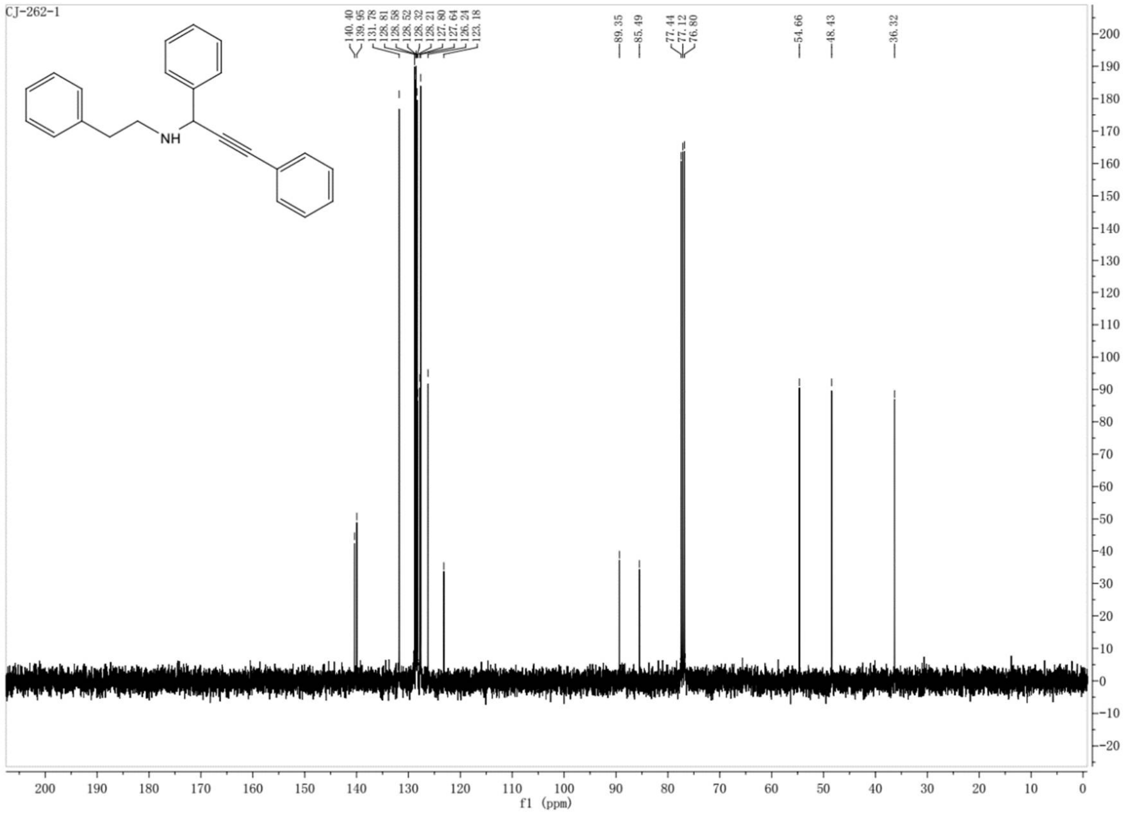

Figure S10. ^{13}C NMR (100 MHz, $CDCl_3$) spectrum of *N*-phenethyl-1,3-diphenylprop-2-yn-1-amine (4d).

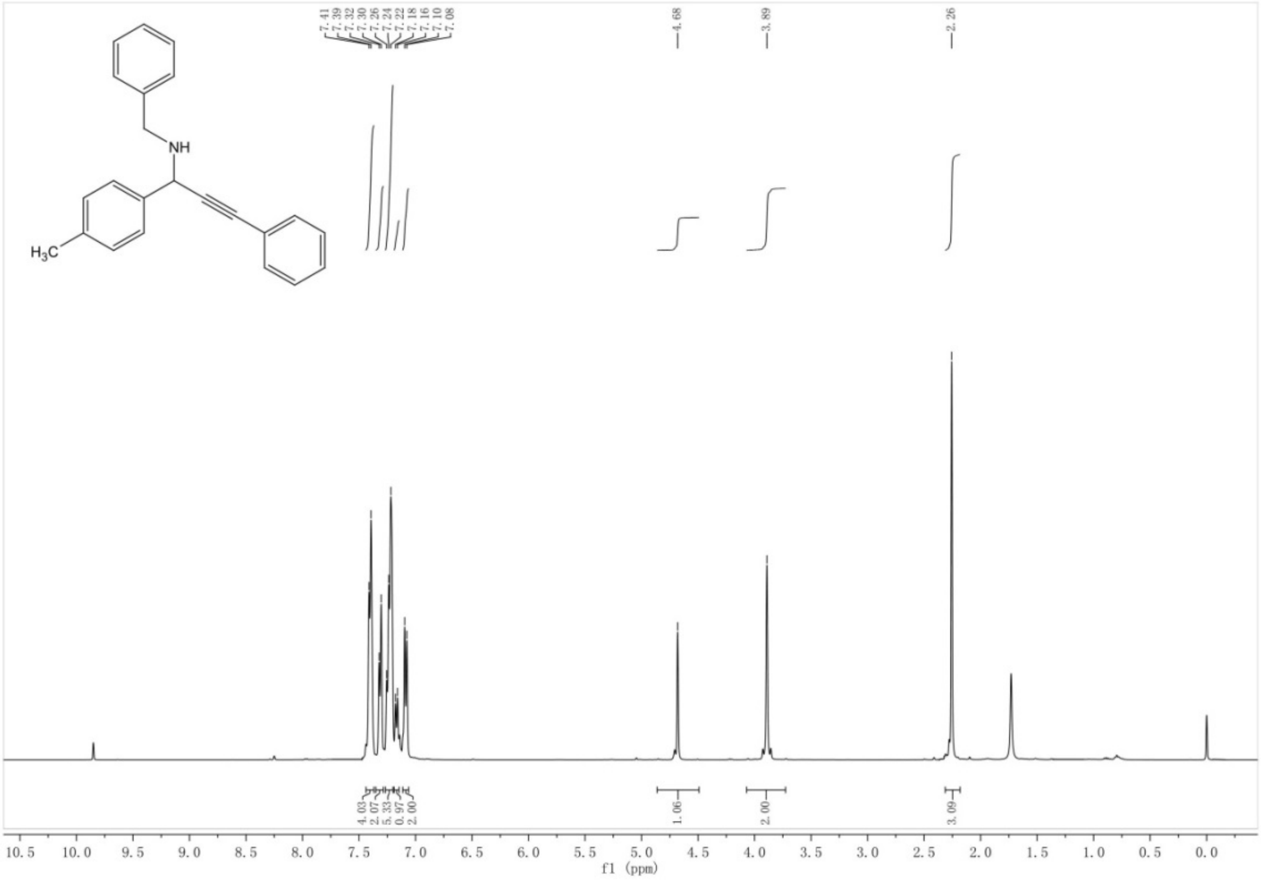

Figure S11. ^{1}H NMR (400 MHz, $CDCl_3$) spectrum of *N*-benzyl-3-phenyl-1-(p-tolyl)prop-2-yn-1-amine (4e).

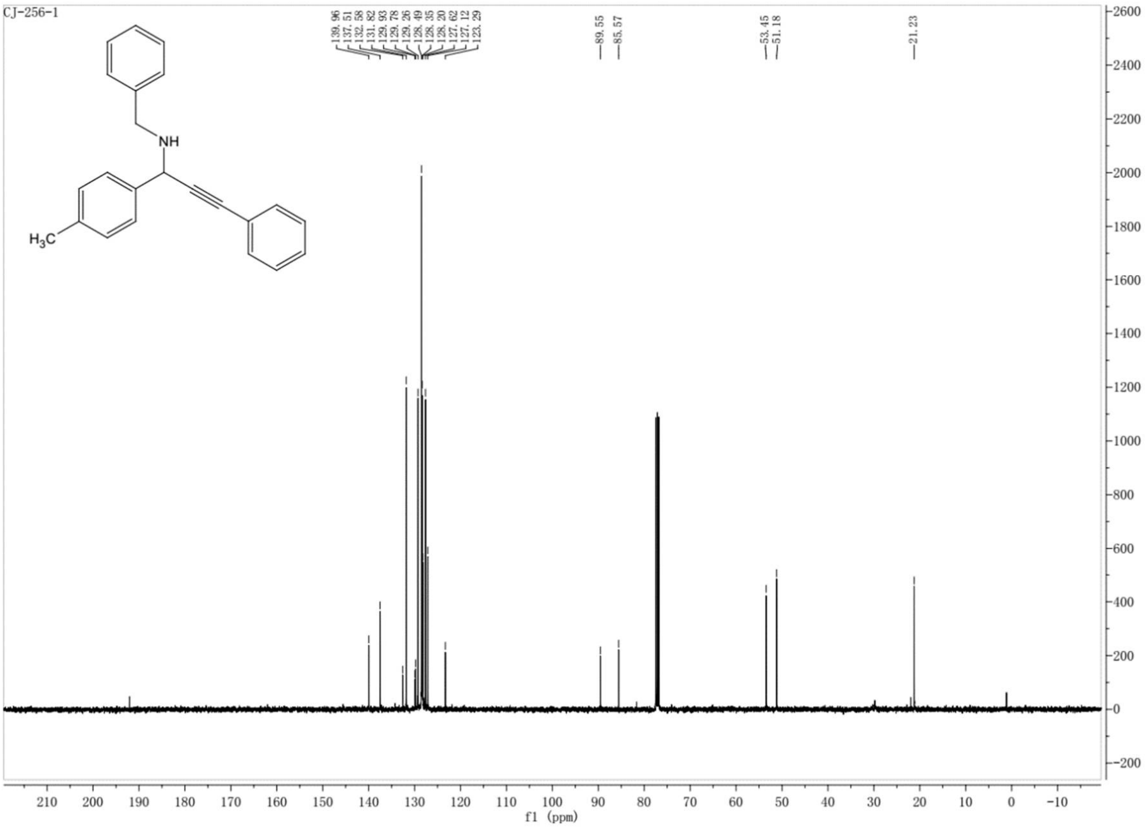

Figure S12. ^{13}C NMR (101 MHz, $CDCl_3$) spectrum of *N*-benzyl-3-phenyl-1-(p-tolyl)prop-2-yn-1-amine (4e).

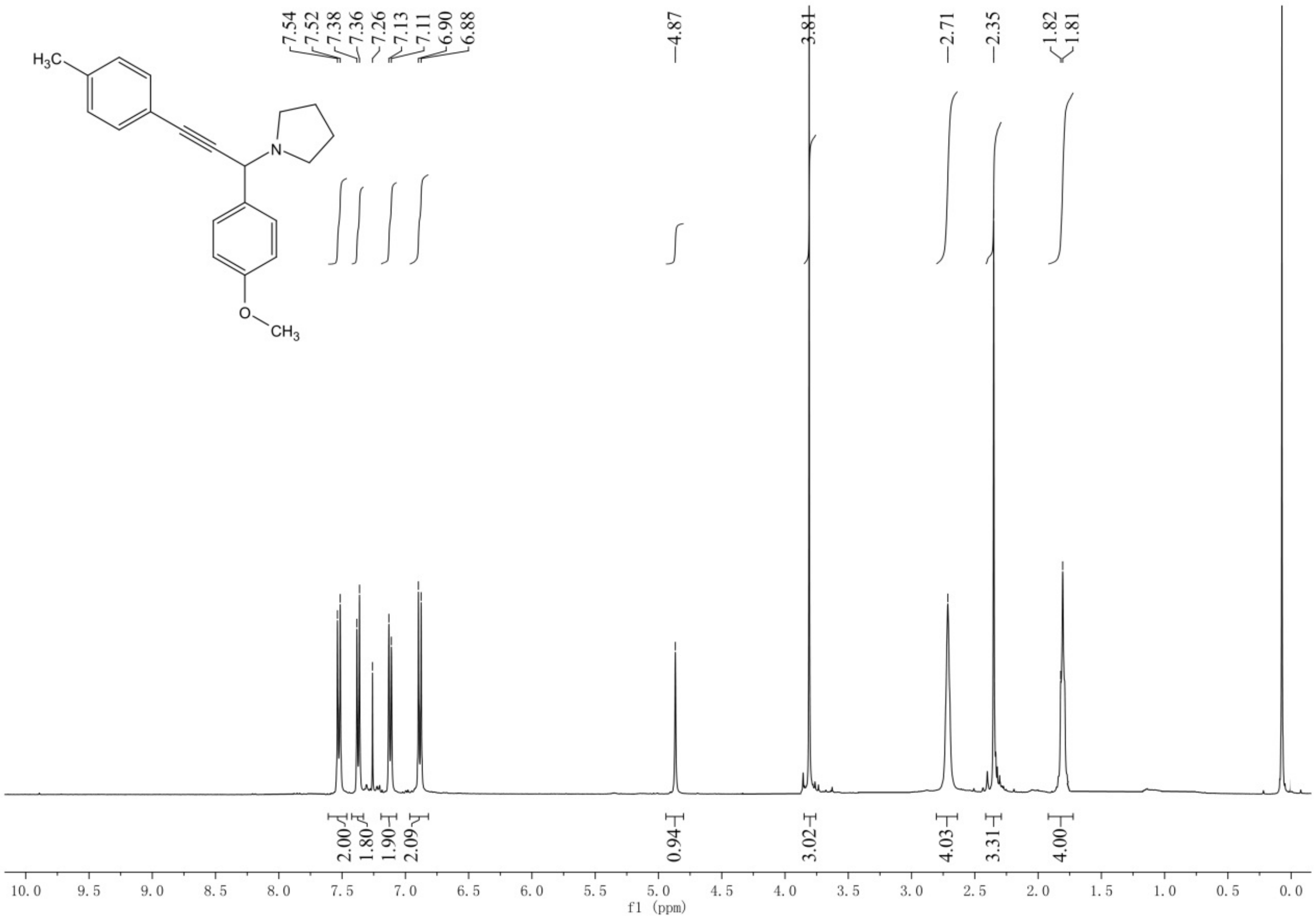

Figure S13. ^{1}H NMR (400 MHz, $CDCl_3$) spectrum of 1-(1-(4-methoxyphenyl)-3-(4-methylphenyl)-2-propynyl)pyrrolidine (4f).

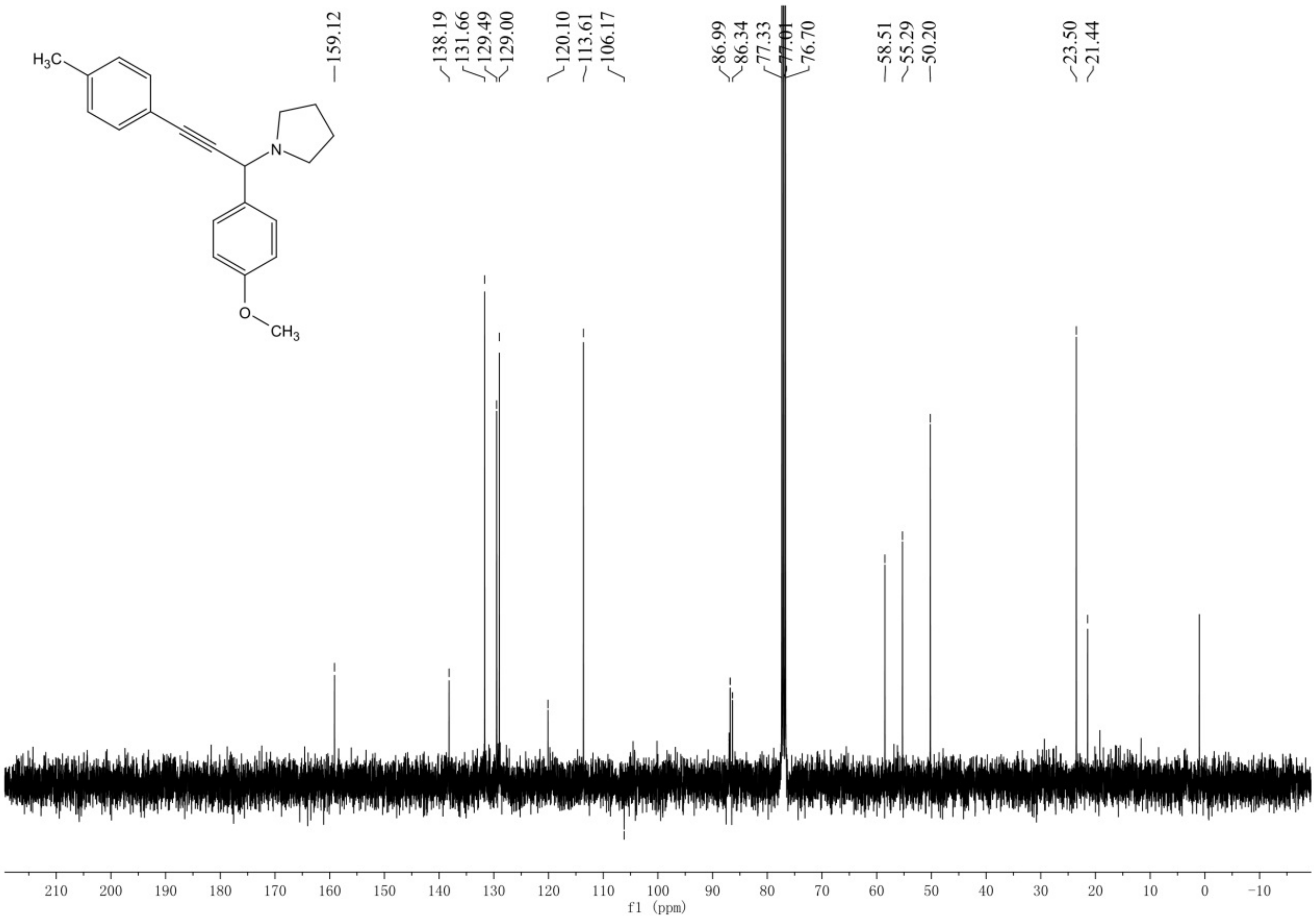

Figure S14. ^{13}C NMR (100 MHz, $CDCl_3$) spectrum of 1-(1-(4-methoxyphenyl)-3-(4-methylphenyl)-2-propynyl)pyrrolidine (4f)).

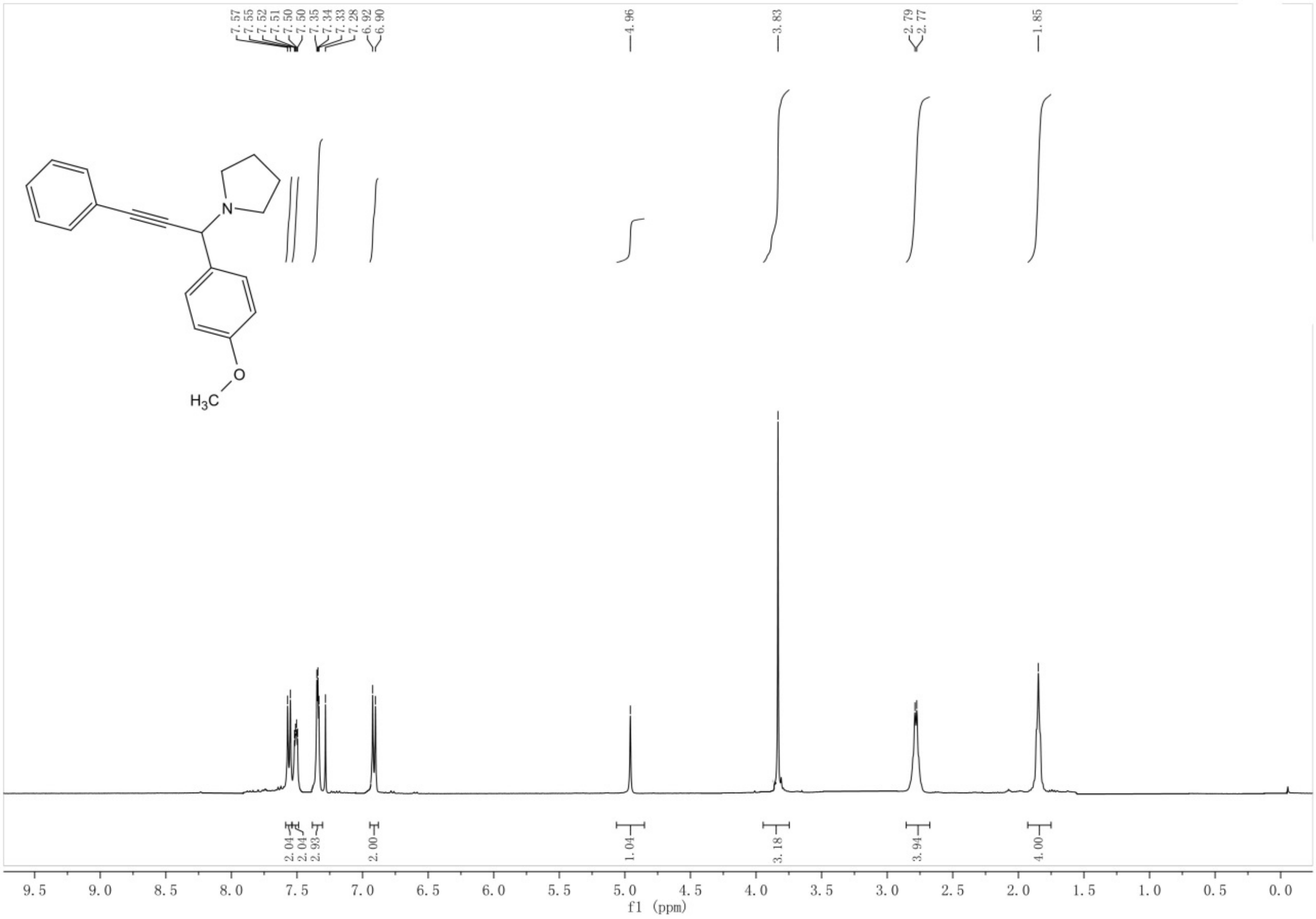

Figure S15. ^{1}H NMR (400 MHz, $CDCl_3$) spectrum of 1-(1-(4-methoxyphenyl)-3-phenyl-2-propynyl)pyrrolidine (4g).

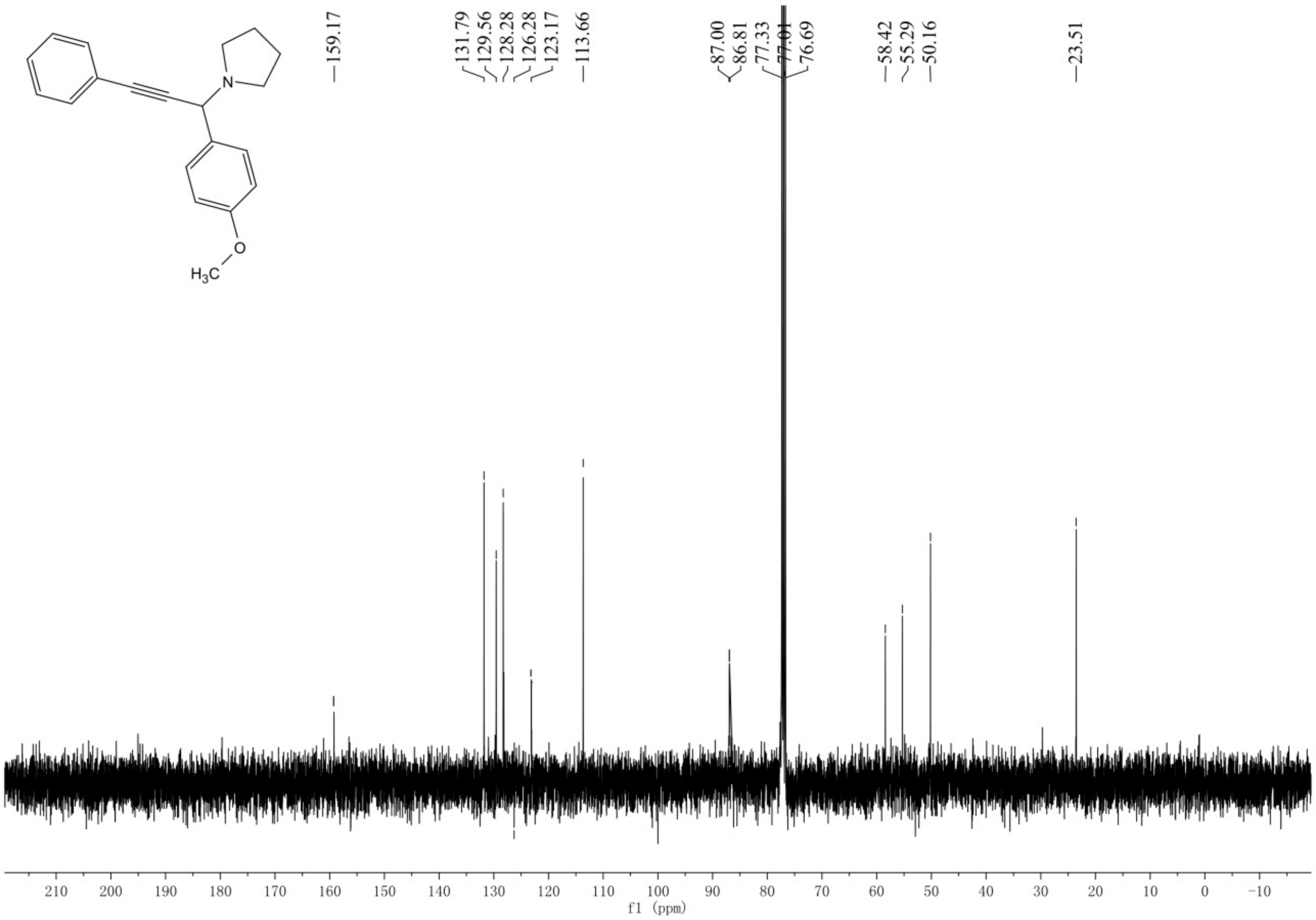

Figure S16. ^{13}C NMR (100 MHz, $CDCl_3$) spectrum of 1-(1-(4-methoxyphenyl)-3-phenyl-2-propynyl)pyrrolidine (4g).

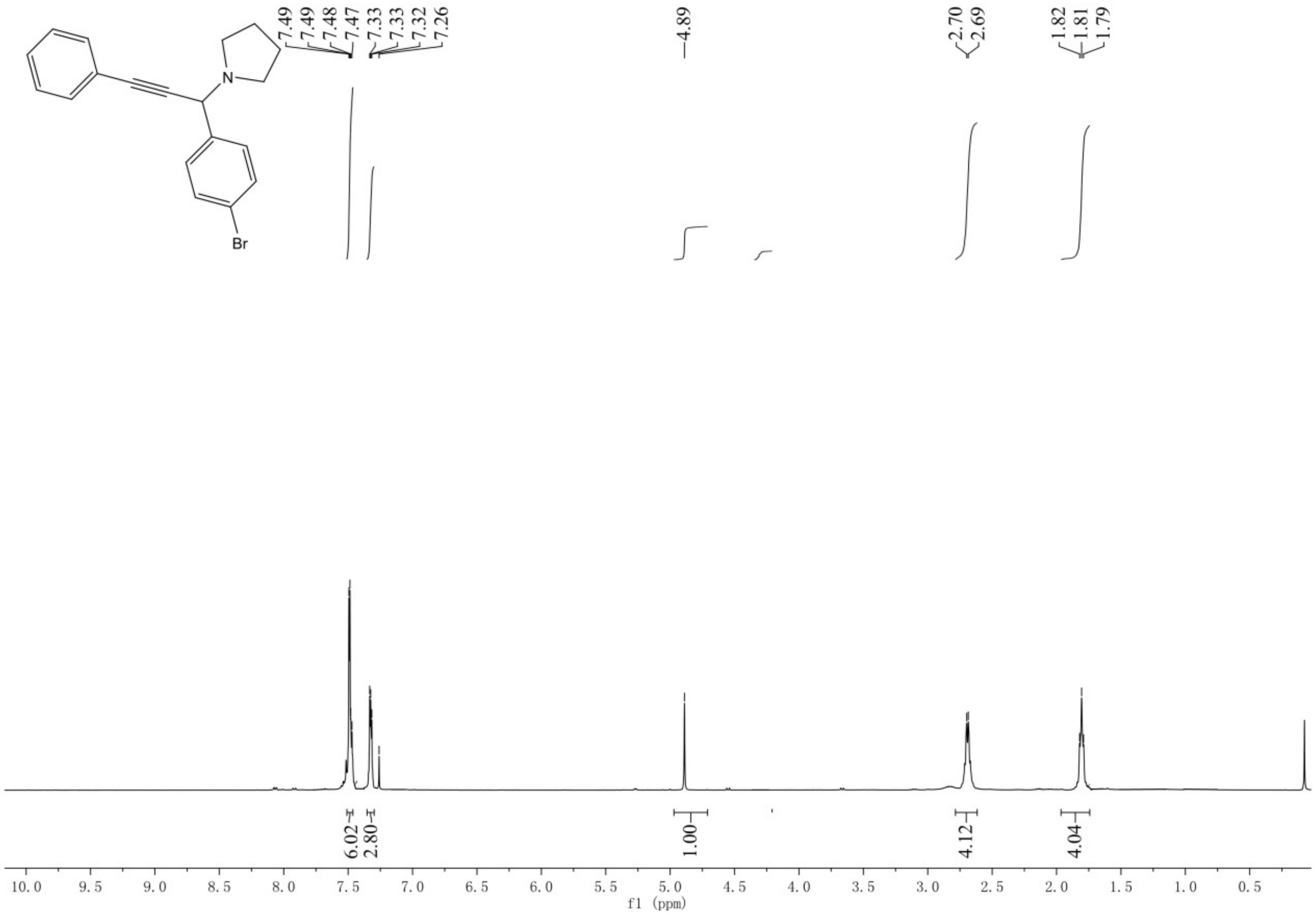

Figure S17. ^{1}H NMR (400 MHz, $CDCl_3$) spectrum of 1-(1-(4-bromophenyl)-3-phenyl-2-propynyl)pyrrolidine (4h).

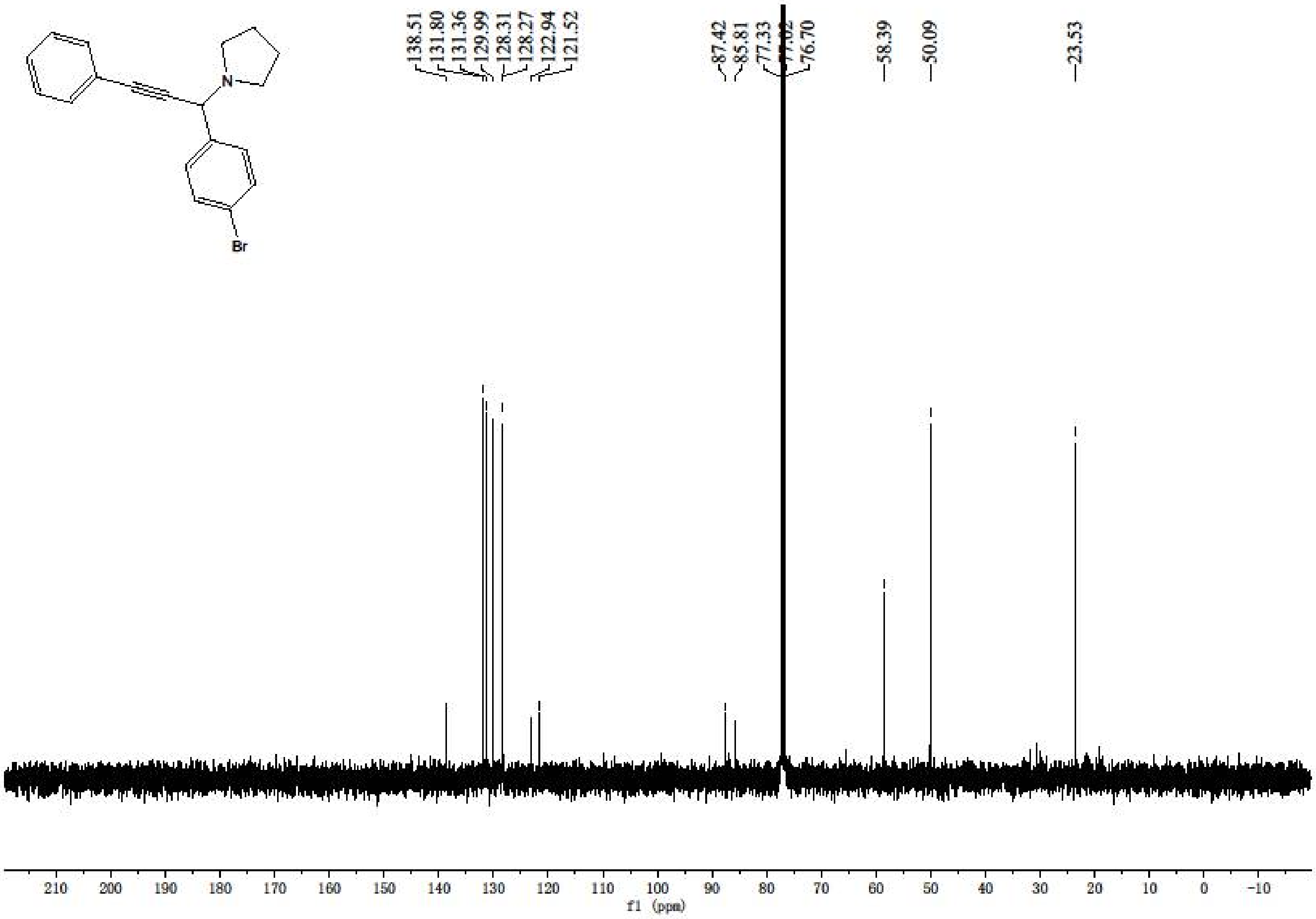

Figure S18. ^{13}C NMR (100 MHz, $CDCl_3$) spectrum of 1-(1-(4-bromophenyl)-3-phenyl-2-propynyl)pyrrolidine (4h).

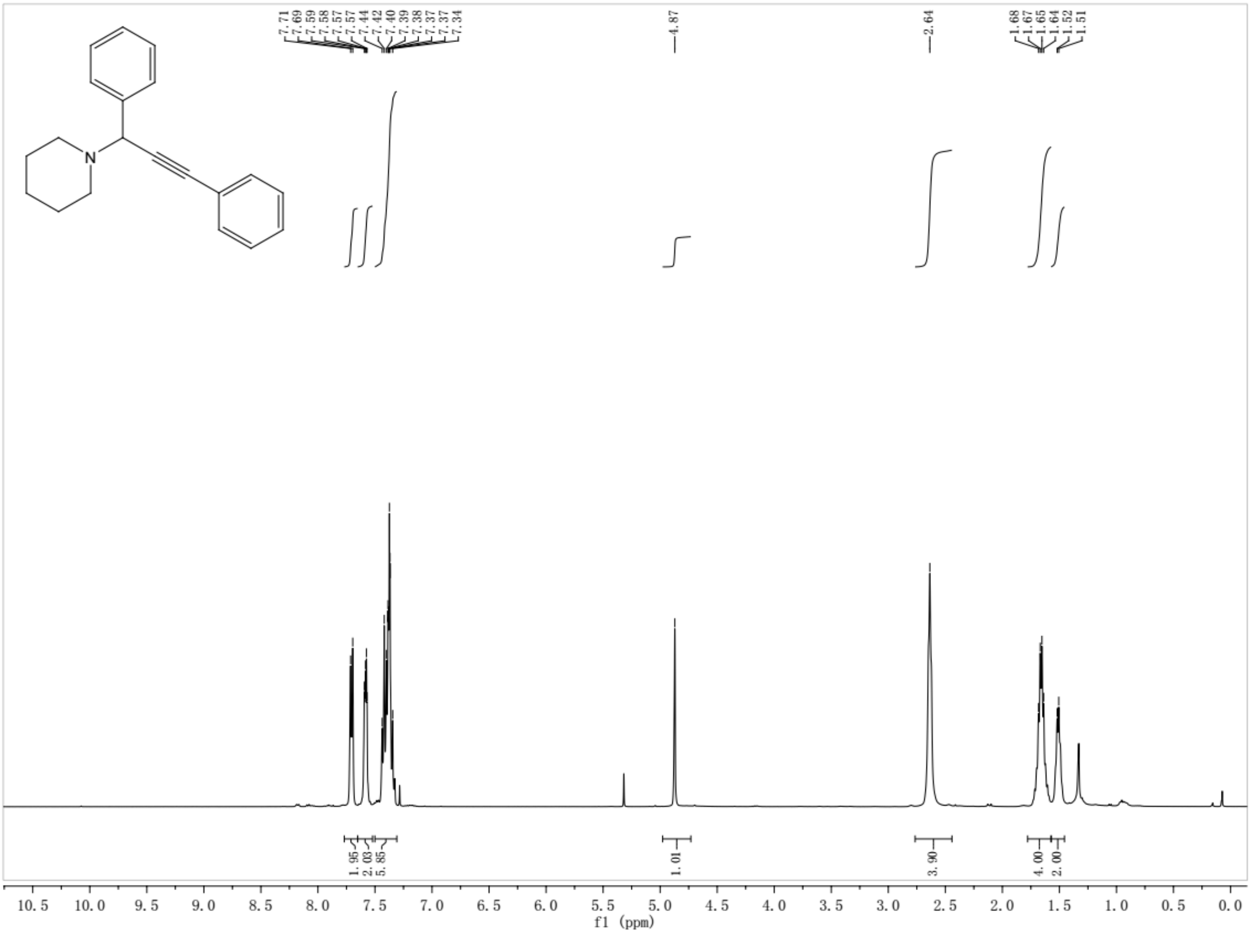

Figure S19. ^{1}H NMR (400 MHz, $CDCl_3$) spectrum of 1-(1,3-diphenyl-2-propynyl)pyrrolidine (4i).

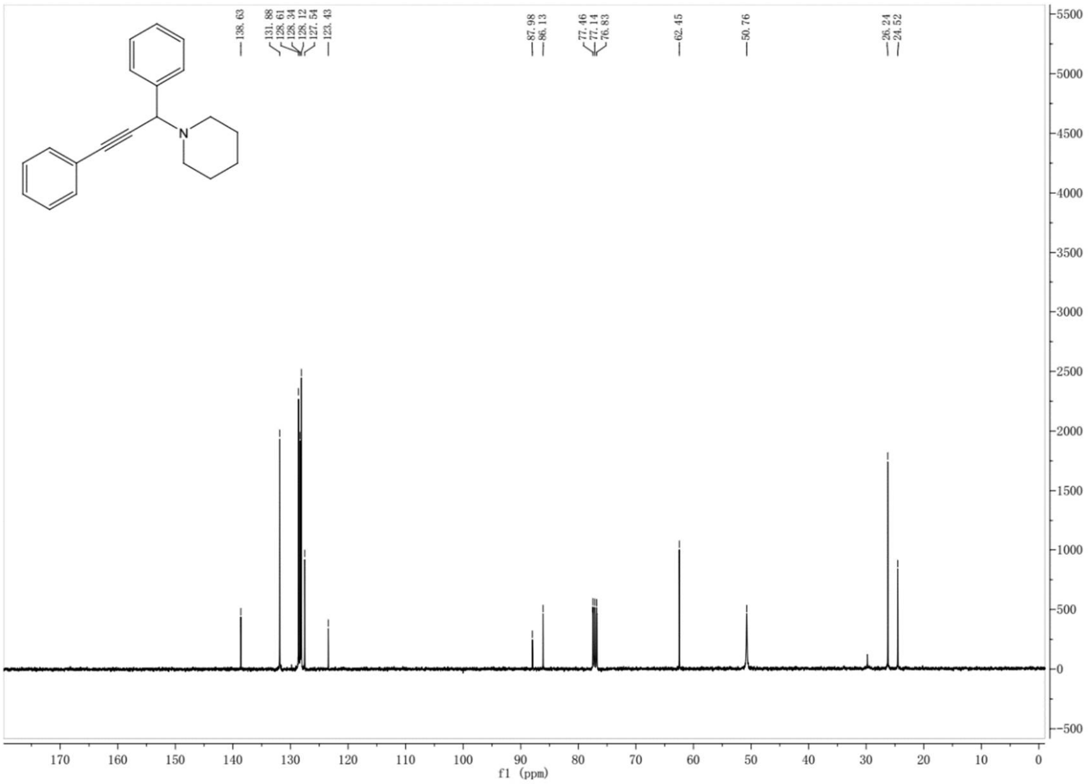

Figure S20. ^{13}C NMR (101 MHz, $CDCl_3$) spectrum of 1-(1,3-diphenyl-2-propynyl)pyrrolidine (4i).

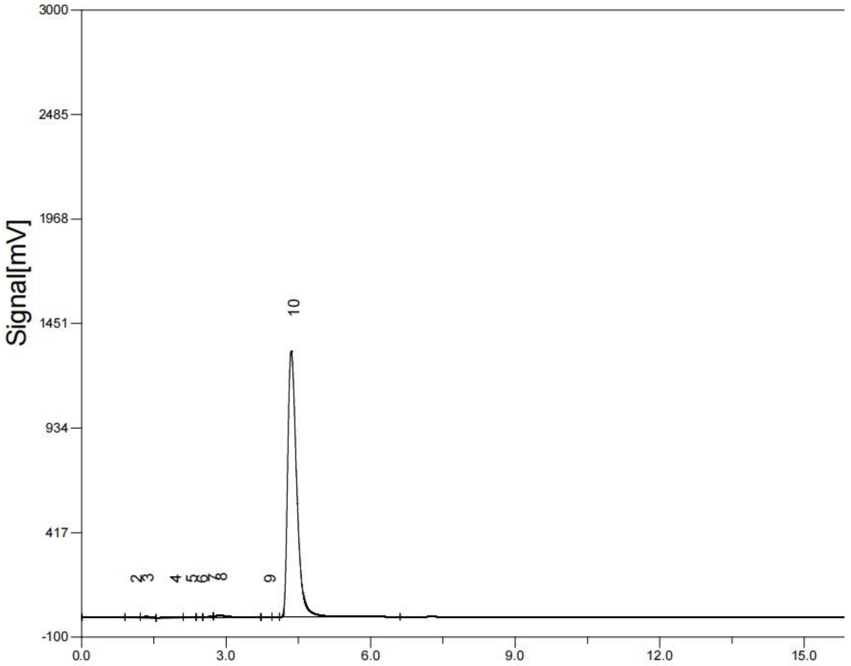

Racemic.

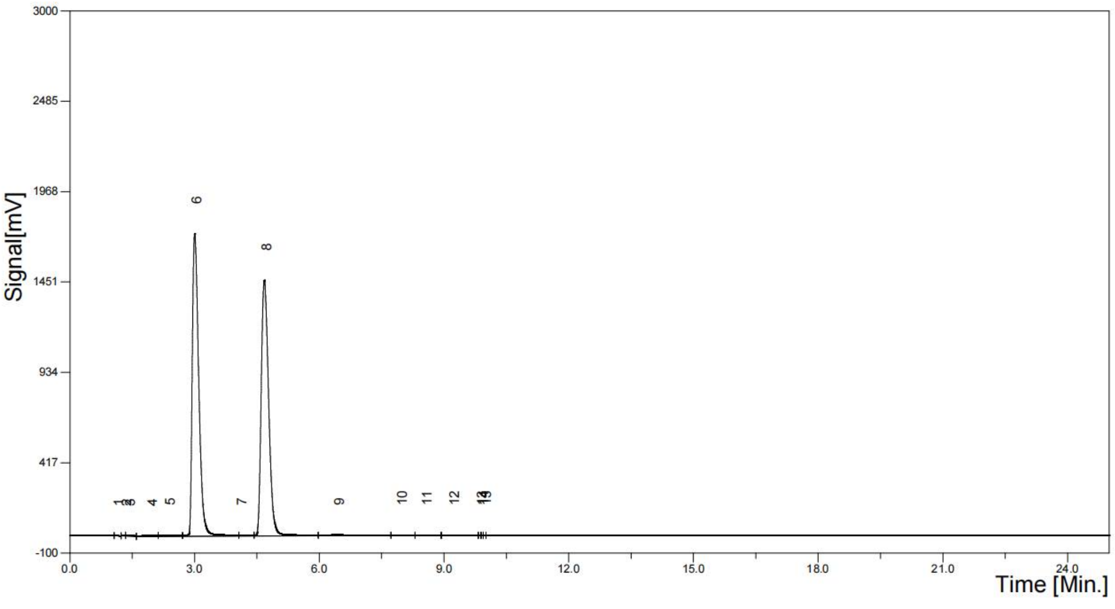

Figure S21. HPLC Profile of 4a.

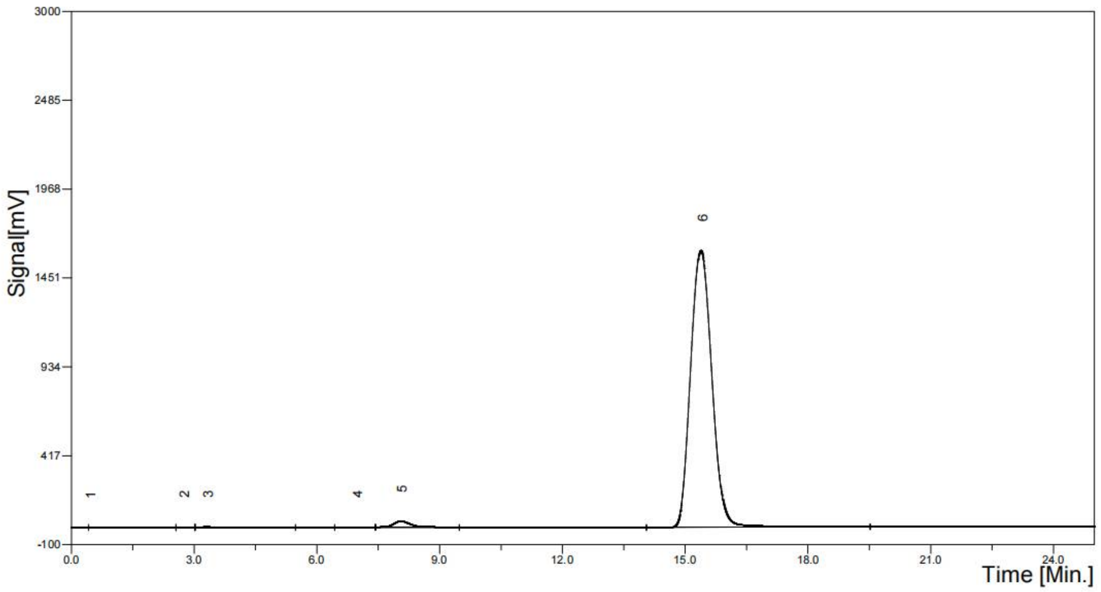

Racemic.

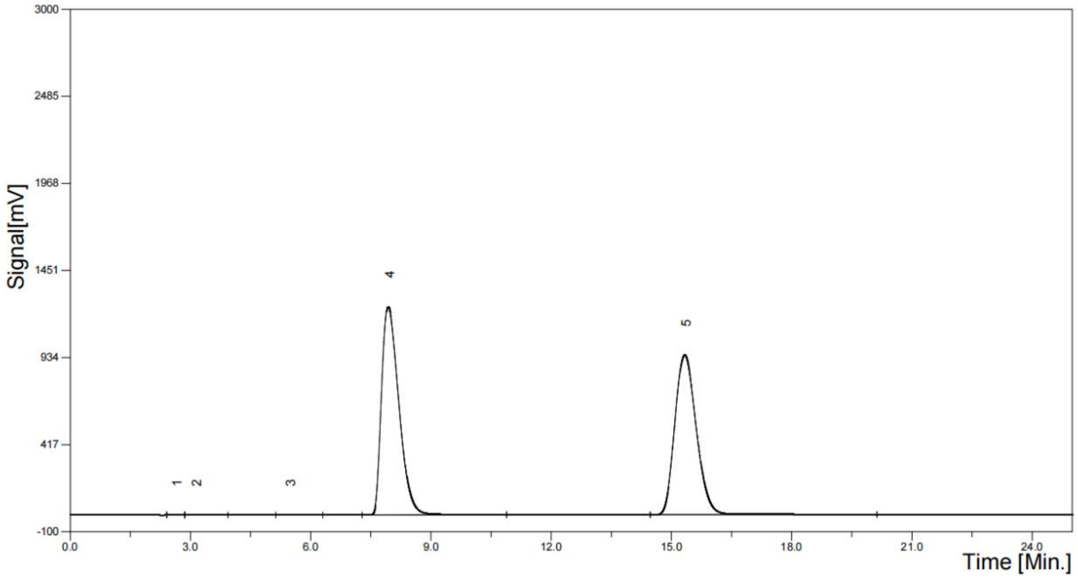

Figure S22. HPLC Profile of 4b.

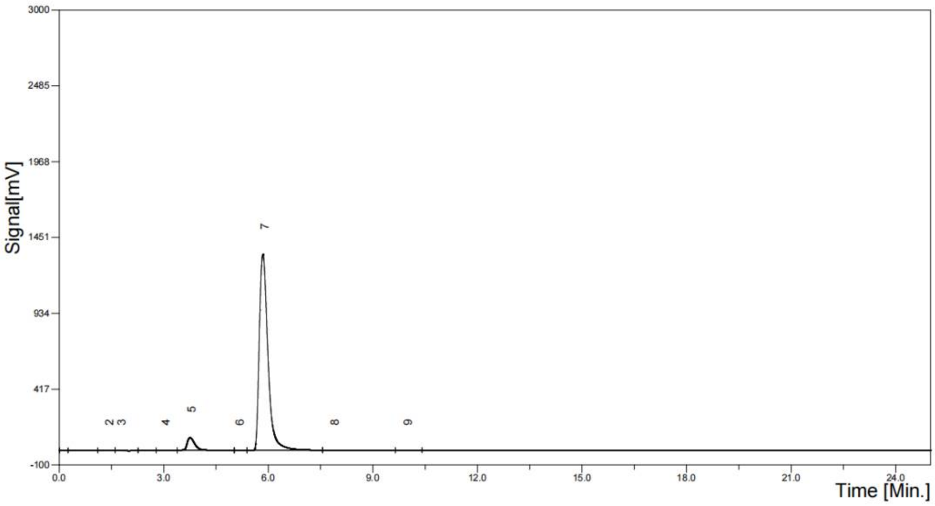

Racemic.

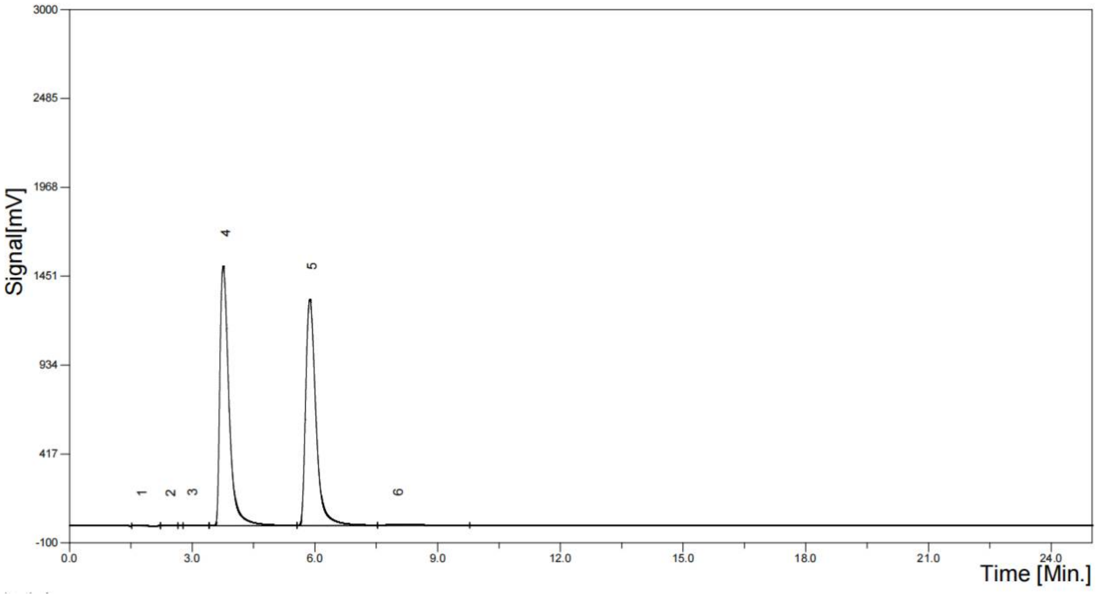

Figure S23. HPLC Profile of 4c.

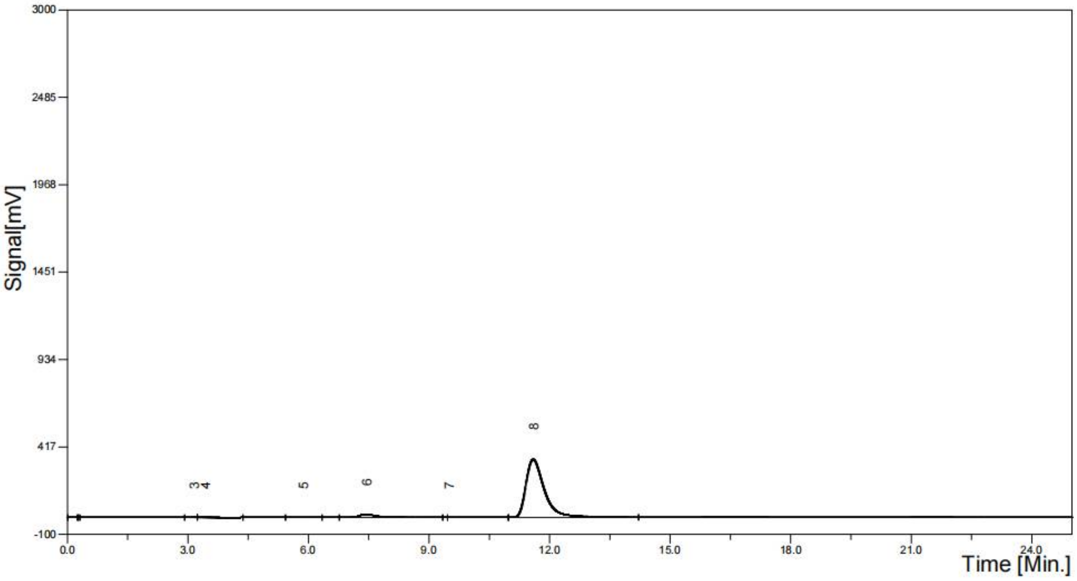

Racemic.

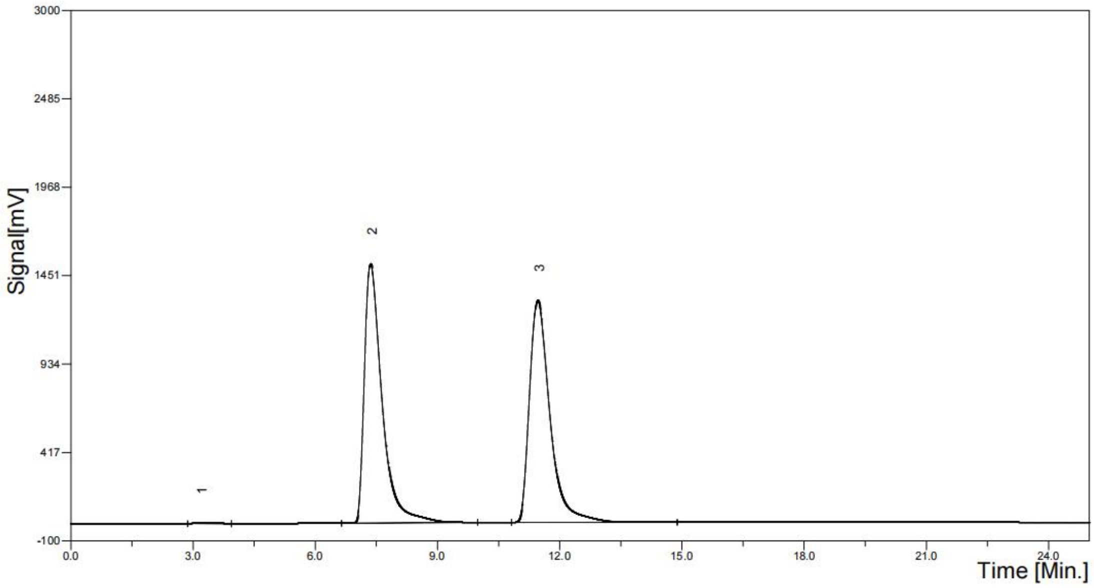

Figure S24. HPLC Profile of 4d.

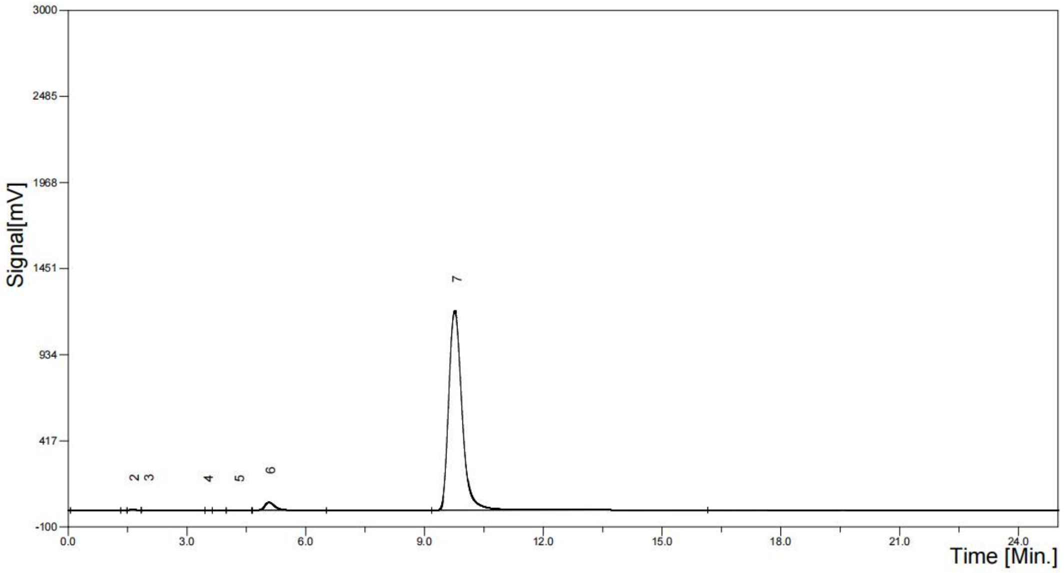

Racemic.

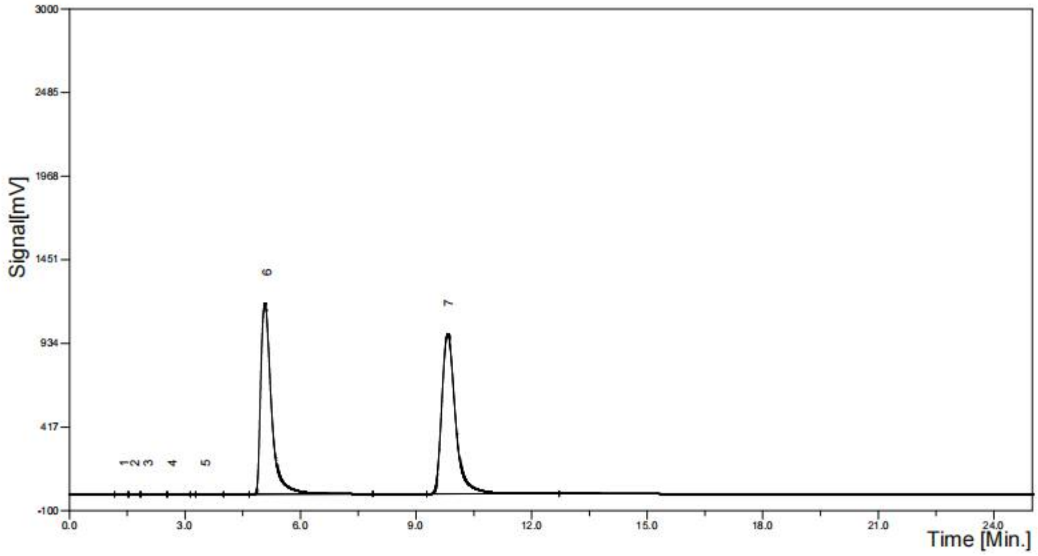

Figure S25. HPLC Profile of 4e.

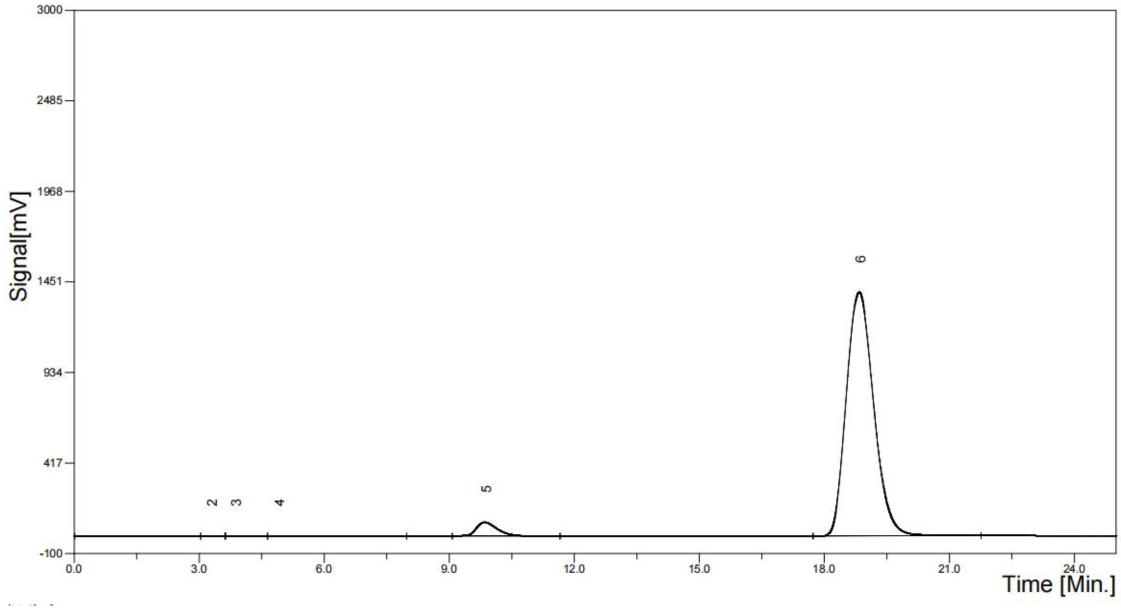

Racemic.

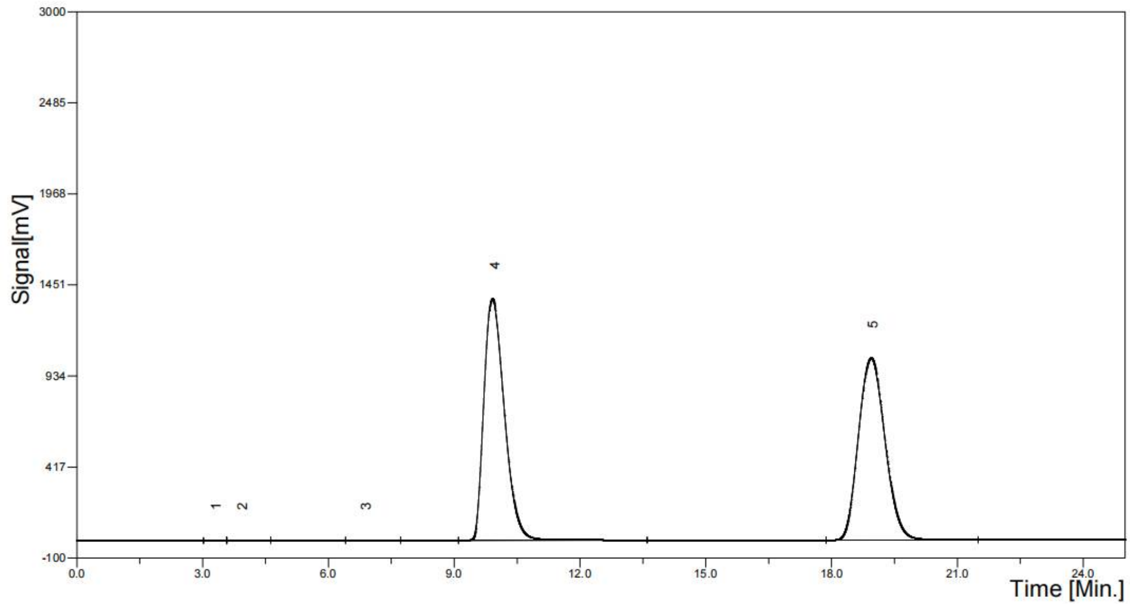

Figure S26. HPLC Profile of 4f.

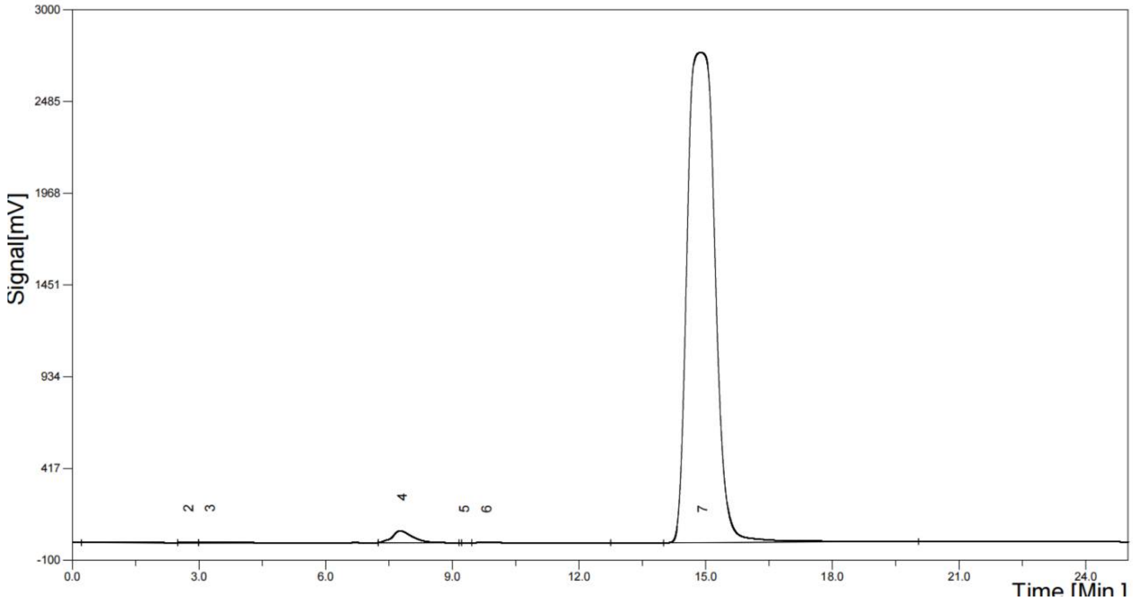

Racemic.

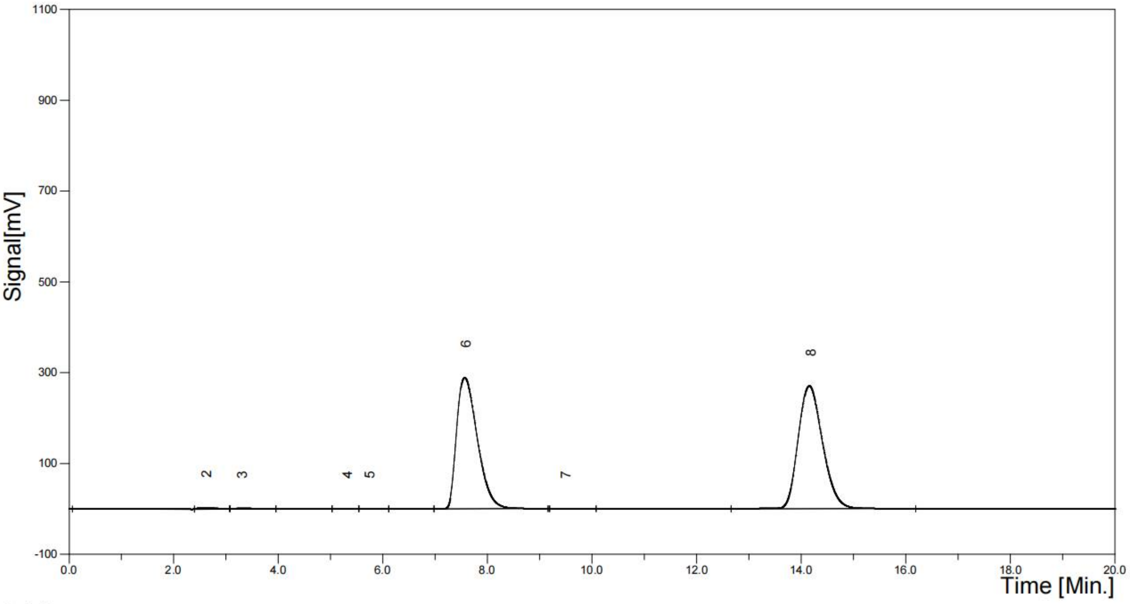

Figure S27. HPLC Profile of 4g.

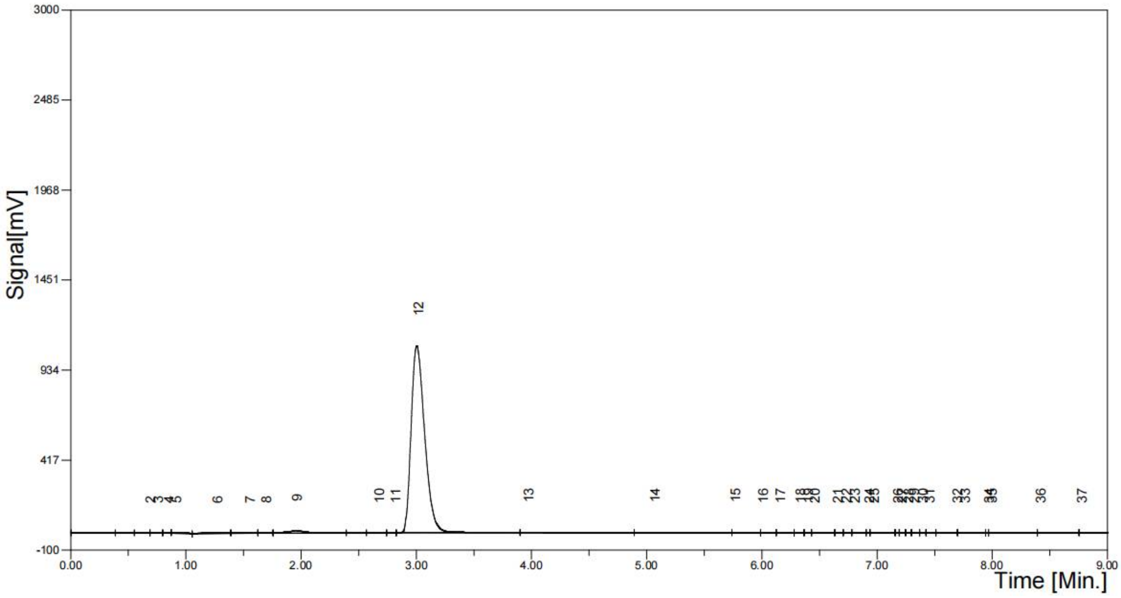

Racemic.

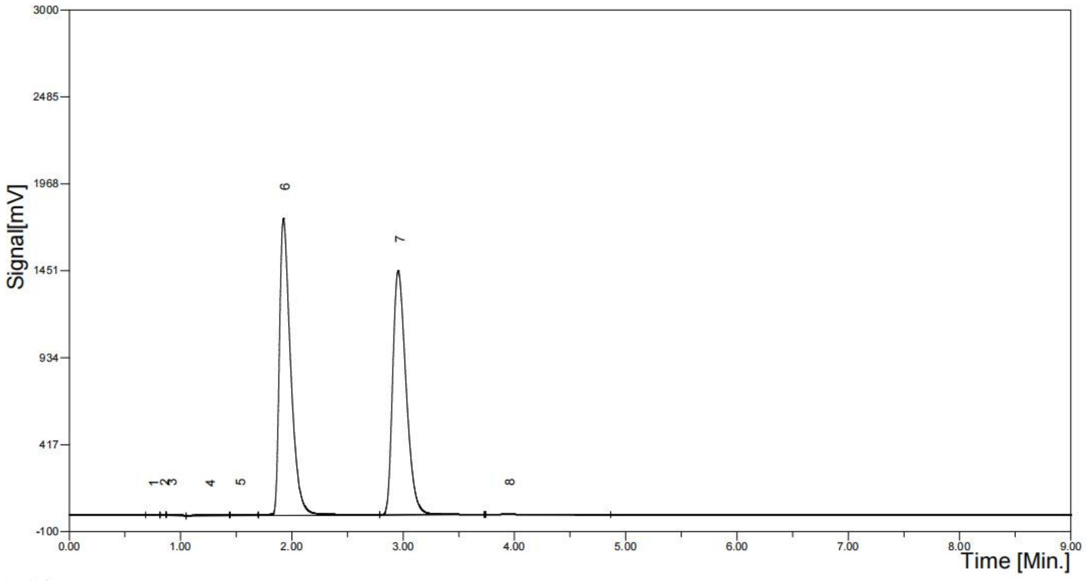

Figure S28. HPLC Profile of 4h.

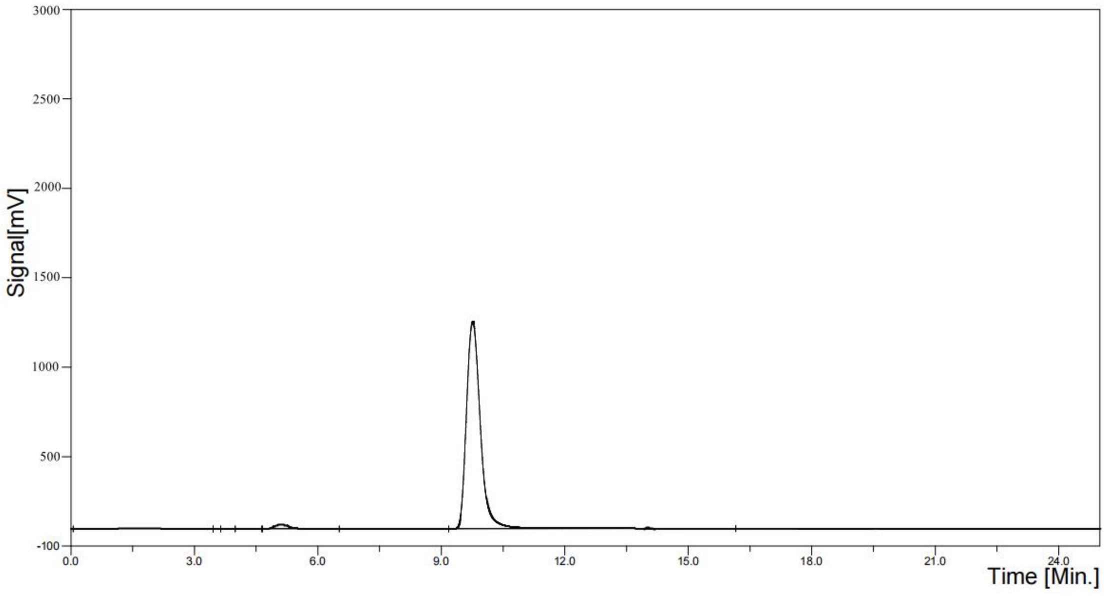

Racemic.

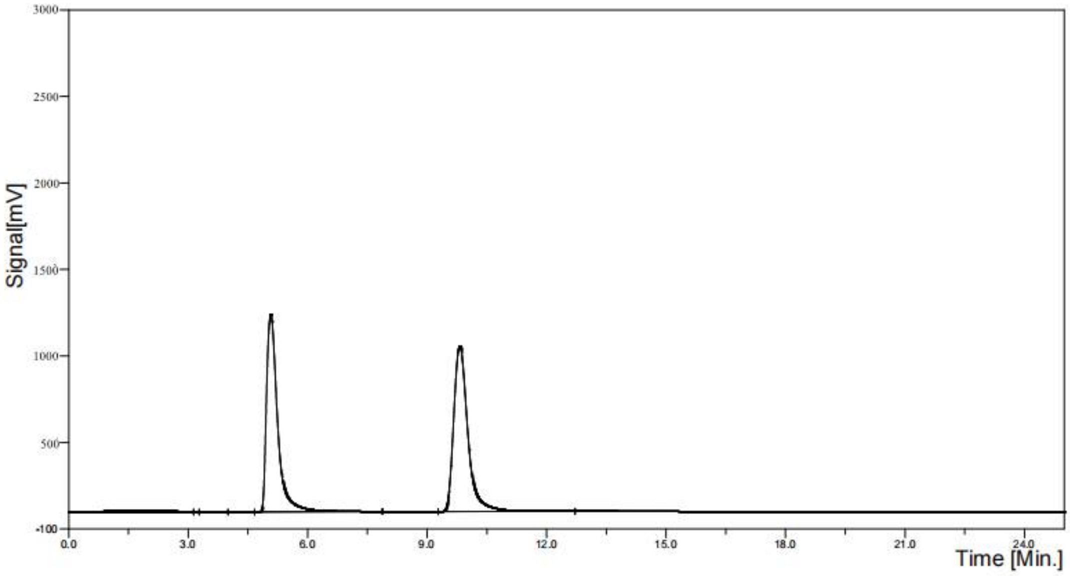

Figure S29. HPLC Profile of 4i.

References

1. Zhao, C.; Seidel, D. Enantioselective a(3) Reactions of Secondary Amines with a Cu(I)/Acid-Thiourea Catalyst Combination. *Journal of the American Chemical Society*, **2015**, *137*, 4650–4653.

2 Zhaokun Li, Feng Zhao, Wei Ou, Pei-Qiang Huang, and Xiaoming Wang, 'Asymmetric Deoxygenative Alkynylation of Tertiary Amides Enabled by Iridium/Copper Bimetallic Relay Catalysis', *Angewandte Chemie International Edition*, 60 (2021), 26604-09.

3 Yingdong Lu, Tim C. Johnstone, and Bruce A. Arndtsen, 'Hydrogen-Bonding Asymmetric Metal Catalysis with A-Amino Acids: A Simple and Tunable Approach to High Enantioinduction', *Journal of the American Chemical Society*, 131 (2009), 11284-85.
